# Unveiling the Role of circRNAs in Pyroptotic Signalling: From Molecular Crosstalk to Disease Modulation

**DOI:** 10.1111/jcmm.70954

**Published:** 2025-11-24

**Authors:** Tengyu Jin, Guodong Xu, Wanru Zhou, Yige Shi, Hebo Wang

**Affiliations:** ^1^ Hebei Medical University Shijiazhuang China; ^2^ Hebei General Hospital Affiliated to Hebei Medical University Shijiazhuang China; ^3^ Hebei Provincial Key Laboratory of Cerebral Networks and Cognitive Disorders Shijiazhuang China; ^4^ Breast Center The Fourth Hospital of Hebei Medical University Shijiazhuang China

**Keywords:** circular RNAs, inflammation, pyroptosis, tumour

## Abstract

Pyroptosis is a gasdermins‐dependent programmed cell death (PCD) characterised by progressive cellular swelling and plasma membrane rupture (PMR). This process releases intracellular contents that amplify inflammatory cascades and immune activation, involving the pathogenesis of various disorders such as tumours, heart and vascular diseases, diabetic complications and inflammatory/infectious disorders. With the advancement of research, the regulatory role of noncoding RNA (ncRNA) in the pyroptosis pathway was delineated. Among, studies have demonstrated that circular RNAs (circRNAs) regulate the pyroptosis cascade mainly through three principal mechanisms: functioning as miRNA sponges, modulating protein activity and encoding functional polypeptides. Numerous circRNAs regulating pyroptosis have been characterised, indicating their significant role in this process and associated disease progression. This review systematically summarised current knowledge on the regulatory mechanisms of circRNAs in canonical, noncanonical and caspase‐3/8‐mediated pyroptosis pathways. We further discussed their pathophysiological roles in disease development and potential clinical applications, aiming to advance mechanistic understanding, facilitate clinical translation and inform diagnostic and therapeutic strategies.

## 1 Introduction

Programmed cell death (PCD) is a genetically regulated process by which multicellular organisms maintain homeostasis and morphogenesis [1]. It can be triggered by programmed suicide mechanisms including apoptosis, necroptosis or dysregulated metabolic pathways, such as ferroptosis [1] and cuproptosis [2]. Among them, pyroptosis, a gasdermin‐mediated form of PCD [3], is characterised by cellular swelling, membrane blebbing with bubble‐like protrusions [4] and nuclear alterations including DNA damage and chromatin condensation [5]. The gasdermin family is currently composed of six paralogous genes in humans (GSDMA/B/C/D, GSDME/DFNA5 and DFNB59/DPJVK) and five in mice (Gsdma1/2/3, Gsdmc1/2/3/4, Gsdmd, Dfna5 and Dfnb59) [6]. Except for DPJVK, all of these members consist of two conserved domains, the GSDM^NT^ pore‐forming domain (PFD) and the GSDM^CT^ repressor domain (RD) [7, 8]. Gasdermin family proteins undergo proteolytic cleavage by activated caspases (caspase‐1 [9], 4/5/11 [10] and 3 [11, 12]/8 [13]) and granzyme [14, 15] in response to extracellular or intracellular stimuli, such as pathogens (e.g., bacteria, viruses), cytotoxic agents and chemotherapy drugs. Cleavage releases the PFD from the RD, enabling PFD oligomerisation and formation of 10–14 nm membrane pores. These pores disrupt the electrochemical gradient, facilitating extracellular release of mature IL‐1β (4.5 nm), IL‐18 (4.5 nm) and caspase‐1 (7.5 nm) [16]. Concurrently, NINJ1 oligomerisation is activated, driving complete plasma membrane rupture (PMR) [17, 18] and liberation of large damage‐associated molecular patterns (DAMPs), such as HMGB1 and S100 proteins. This cascade mediates pyroptotic cell death and amplifies inflammatory signalling [6].

Circular RNAs (circRNAs) were initially detected in plant viroids [19] and were once considered ‘scrambled transcripts’ [20]. Since then, many types of circRNAs, including viral circRNAs as well as other types of circRNAs such as those generated from the noncoding sequences and processed from eukaryotic precursor mRNAs have been identified [21]. However, until 2012, with the application of high‐throughput transcriptome sequencing technology for the detection of total RNAs or nonpolyadenylated RNAs [22, 23], the misperception that circRNAs are lowly presented [24] is gradually being altered. Unlike traditional linear transcripts, circRNAs are a group of covalently closed single‐stranded loops that do not have 5′‐caps and 3′‐poly(A) tails. These circular structures are generated through back‐splicing mechanisms during pre‐mRNA processing [22, 25, 26, 27]. Therefore, circRNAs are distinct from their parent mRNAs in characteristics, biogenesis and wide‐ranging regulatory effects. With the progress of circRNA research, circRNAs have been found to act as dynamic, functional molecules extensively involved in cellular biological, physiological and pathological processes [28]. Emerging evidence highlights the regulatory involvement of circRNAs in modulating key molecular components of the pyroptosis signalling pathway.

In this review, we systematically examine the regulatory roles of circRNAs in multiple pyroptosis pathways and their implications for disease progression, including tumours, heart and vascular diseases, diabetic complications and inflammatory/infectious disorders. Finally, we analyse current challenges and future research directions in elucidating the circRNA–pyroptosis axis and its translational potential for therapeutic development, aiming to facilitate advancements in pyroptosis‐related research and the development of clinical applications.

## 2 Pyroptosis

Pyroptosis is an inflammatory lytic PCD executed through gasdermin‐mediated plasma membrane poration [29]. Specific morphological features include nonapoptotic chromatin condensation and PMR induced by progressive cellular swelling [30]. The term ‘pyroptosis’ was first introduced in 2001 to describe the proinflammatory PCD mechanism that has similarities with apoptosis but depends on inflammatory caspase‐1 activation [31]. Emerging research has revealed an expanding repertoire of pyroptosis pathways beyond the canonical caspase‐1‐dependent mechanism. Human caspase‐4 and caspase‐5 (mouse orthologs caspase‐11) mediate noncanonical pyroptosis through GSDMD cleavage, generating N‐terminal fragments (N‐GSDMD) that execute plasma membrane pore formation [10, 32]. Furthermore, caspase‐3 [11, 12]/8 [33, 34]‐dependent pathways as well as granzyme‐mediated mechanisms have been systematically elucidated, revealing the complexity of pyroptosis.

**FIGURE 1:**
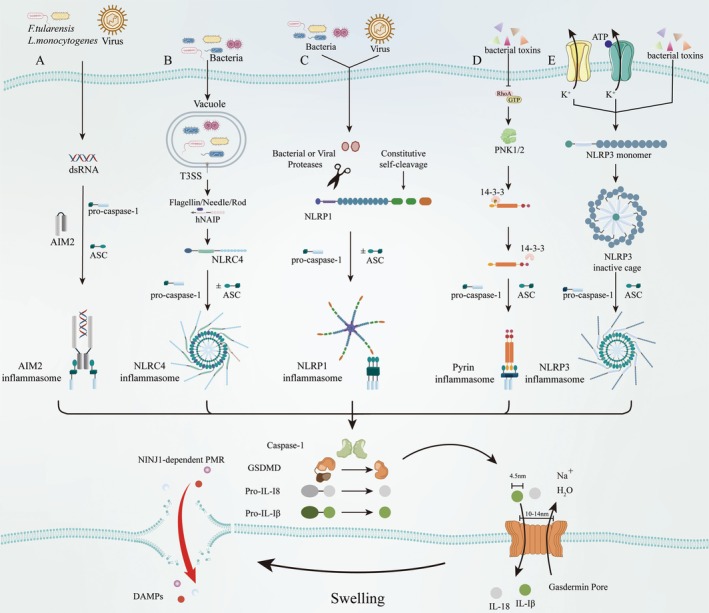
Molecular mechanism of the canonical pyroptosis pathway. (A) AIM2 inflammasome activation pathway. Upon stimulation by bacterial or viral dsDNA, AIMs undergo oligomerisation and subsequently associate with procaspase‐1 through an ASC‐dependent mechanism. (B) NLRC4 inflammasome activation pathway. Activation of the NLRC4 inflammasome requires hNAIP‐mediated recognition of flagellin, needle proteins and the rod component of bacterial T3SS. (C) NLRP1 inflammasome activation pathway. In the presence of bacterial or viral proteases, the NLRP1 inhibitory domain undergoes proteolytic degradation and subsequent activation. It subsequently assembles with procaspase‐1 in an ASC‐dependent or ASC‐independent manner to form an inflammasome. (D) Pyrin inflammasome activation pathway. Following RhoA GTPase inactivation by stimuli such as bacterial toxins, the PKN1/2‐mediated phosphorylation of pyrin is impaired, leading to its dissociation from the inhibitory 14‐3‐3 proteins and subsequent activation. (E) NLRP3 inflammasome activation pathway. In its autoinhibited state, NLRP3 is in a cage conformation. Multiple stimuli—including potassium (K^+^) efflux, the bacterial toxin nigericin, gout‐associated urate crystals and extracellular ATP—induce NLRP3 activation and organise into a decameric inflammasome complex. Created in Adobe Illustrator 2025.

Canonical pathway is mediated through inflammasomes assembly accompanied by caspase‐1 activation, GSDMD cleavage and subsequent secretion of IL‐1β and IL‐18 [35]. This pathway is initiated by cytosolic pattern recognition receptors (PRRs, also termed inflammasome sensors) upon detection of pathogen‐associated molecular patterns (PAMPs) and DAMPs. Certain ‘sensor’ proteins are directly activated by DAMPs or PAMPs, while others function as indirect detectors of cellular disturbances induced by these molecular patterns. The first group can be categorised into two distinct categories based on their activation mechanisms. The first category comprises absent in melanoma 2 (AIM2), which recognises viral or bacterial dsDNA through its C‐terminal HIN200 domain (Figure 1A) [36, 37], and NOD‐like receptor family apoptosis inhibitory proteins (NAIPs) that specifically bind bacterial flagellin, needle and Bacterial Type III secretion systems (T3SS) rod components [38, 39]. This specific ligand recognition triggers NLR family CARD domain‐containing 4 (NLRC4) inflammasome activation, resulting in caspase‐1 cleavage and subsequent pyroptosis (Figure 1B) [40]. The second activation mechanism involves NOD‐like receptor family pyrin domain‐containing protein 1 (NLRP1) [41], which undergoes direct enzymatic modification by bacterial or viral proteases, resulting in proteolytic degradation of its inhibitory domain and subsequent inflammasome activation [41, 42]. Structurally, the C‐terminal domain of NLRP1 contains a caspase recruitment domain (CARD)‐binding motif that facilitates caspase‐1 activation through CARD‐CARD interaction with procaspase‐1 (Figure 1C) [43, 44]. Sensors indirectly monitoring PAMPs or DAMPs include PYRIN and NLRP3. PYRIN is activated following Rho GTPase inactivation triggered by bacterial toxins such as *Clostridium difficile* glycosyltransferase TcdB and *Vibrio parahaemolyticus* VopS et al. Disruption of RhoA function impairs PKN1/2‐mediated phosphorylation of pyrin, thereby inducing its dissociation from the inhibitory 14–3‐3 proteins and subsequent activation (Figure 1D) [45], while NLRP3 responds to diverse stimuli including the potassium (K^+^) efflux, bacterial toxin nigericin, gout‐associated uric acid crystals and extracellular ATP (Figure 1E) [46, 47]. Notably, both AIM2, PYRIN and NLRP3 activate caspase‐1 through an apoptosis‐associated speck‐like protein containing a CARD (ASC)‐dependent mechanism [1]. The activated caspase‐1 specifically cleaves the executor protein GSDMD at Asp275, producing a 22‐kDa C‐GSDMD and a 31‐kDa N‐GSDMD. The N‐GSDMD oligomerises to create nonselective plasma membrane pores (10–14 nm diameter), which disrupt osmotic homeostasis, culminating in NINJ1‐dependent PMR and subsequent release of substantial quantities of proinflammatory DAMPs [16, 17]. Concurrently, caspase‐1 processes pro‐IL‐1β and pro‐IL‐18 into their mature forms [6]. These inflammatory cytokines are subsequently released through GSDMD‐formed pores, thereby inducing pyroptosis [48].

**FIGURE 2:**
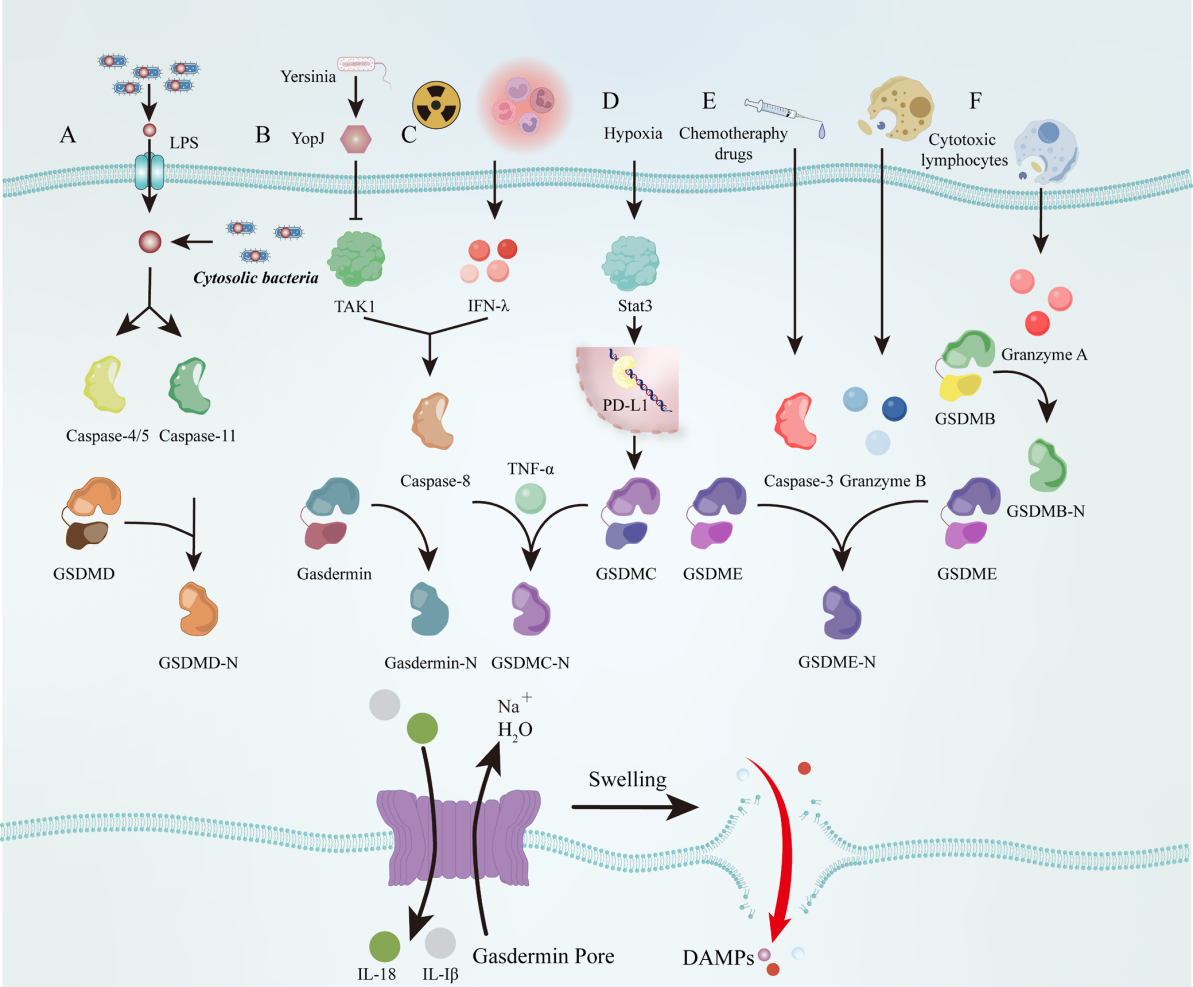
Molecular mechanism of noncanonical, caspase‐3/8‐mediated and granzyme‐mediated pyroptosis pathway. (A) Noncanonical pyroptosis pathway. Direct LPS engagement activates caspase‐4/5 (caspase‐11 in mice), triggering GSDMD cleavage, generation of the pore‐forming GSDMD‐N fragment and pyroptosis execution. (B) Caspase‐8/gasdermins‐mediated pyroptosis. *Yersinia* utilises the effector YopJ to suppress TAK1 activity, thereby activating caspase‐8 and inducing proteolytic cleavage of both GSDMD and GSDME, which drives pyroptosis. (C) IFN‐λ induced by colitis or irradiation triggers caspase‐8‐dependent GSDMC‐mediated pyroptosis. (D) Hypoxic conditions enhance the physical interaction between p‐Stat3 and PD‐L1, facilitating PD‐L1 nuclear translocation to upregulate GSDMC transcription. TNF‐α activates caspase‐8, which cleaves GSDMC to execute pyroptosis. (E) Caspase‐3/GSDME‐mediated pyroptosis. Chemotherapeutic drugs induce caspase‐3 activation, triggering GSDME cleavage and subsequent pyroptosis. (F) Killer cells induce target cell pyroptosis through granzyme secretion‐driven gasdermin cleavage. Created in Adobe Illustrator 2025.

In contrast to canonical pyroptosis, the activation of caspase‐4/5 (mouse orthologs caspase‐11) in the noncanonical pathway is mediated through direct interaction with lipopolysaccharide (LPS) [10]. Activated caspase‐4/5/11 cleaves GSDMD to generate its pore‐forming N‐GSDMD, thereby inducing pyroptosis [10]. Similar to caspase‐1, caspases‐4/5 (but not caspase‐11) exhibit the capacity to cleave the identical tetrapeptide cleavage site in pro‐IL‐18 [49] and caspase‐4/5/11 are able to mediate the secretion of IL‐1β and IL‐18 via N‐GSDMD‐generated membrane pores that drive K^+^ efflux (activating the NLRP3 inflammasome) or via the production of an aminoterminal fragment [49, 50, 51, 52, 53] (Figure 2A).

Advancing research in pyroptosis has changed our understanding of PCD: apoptosis‐executing caspases, caspase‐3 [11, 12] and caspase‐8 [33, 34], previously considered incapable of inducing pyroptosis, have now been demonstrated to trigger this lytic inflammatory process. Chemotherapeutic drugs could induce caspase‐3‐mediated GSDME cleavage with high GSDME expression and form N‐GSDME, which executes pyroptosis (Figure 2E) [11, 12]. This PCD is observed in many normal cells but not in most cancer cells, which is attributed to differential GSDME expression profiles between normal and cancer cells [11, 12]. Recently, caspase‐8‐mediated pyroptosis has also been extensively studied [33, 34, 54, 55, 56]. Pathogenic *Yersinia* utilise the effector YopJ to suppress critical immune signalling kinases, including TGF‐β‐activated kinase 1 (TAK1) of the MAP kinase pathway and IKK kinases and activate caspase‐8‐dependent cell death pathways. Notably, during TAK1 inhibition in murine macrophages, caspase‐8 activation initiates proteolytic cleavage of both GSDMD and GSDME, culminating in pyroptosis. Loss of GSDMD shifts the cell death morphology towards apoptotic characteristics, revealing a TAK1/IKK‐dependent regulatory mechanism governing gasdermin processing and cell death modality determination (Figure 2B) [33, 34]. Notably, PD‐L1 is participating in the cell death modality switch in cancer cells, converting TNF‐α‐induced apoptosis to pyroptosis under specific conditions. Hypoxic environments induce physical interaction between p‐Stat3 and PD‐L1, facilitating PD‐L1 nuclear translocation which subsequently enhances GSDMC transcription. TNF‐α stimulation activates caspase‐8, which subsequently mediates cleavage of GSDMC at specific residues, generating the pore‐forming GSDMC‐N. This processed fragment executes pyroptosis through oligomerisation and plasma membrane pore formation (Figure 2D) [55]. In addition, α‐Ketoglutarate (α‐KG) [56] or type III interferons (IFN‐λ) [54] induced by intestinal damage after colitis or irradiation are also capable of triggering caspase‐8/GSDMC‐mediated pyroptosis (Figure 2C).

The traditional perspective that pyroptosis is exclusively mediated by caspases has been challenged by the discovery of granzyme‐dependent proteolytic cleavage of gasdermin proteins. Killer cells induce pyroptosis through a caspase‐independent mechanism wherein granzyme B directly cleaves GSDME at the identical caspase‐3 recognition site in target cells [14] (Figure 2F). Moreover, granzyme A released by cytotoxic lymphocytes executes proteolytic cleavage of GSDMB, triggering its pore‐forming activation and subsequent pyroptosis in GSDMB‐expressing cells. Mechanistic studies reveal this lytic pathway drives robust cytotoxic T lymphocyte (CTL)‐mediated tumour eradication in murine models, establishing the GZMA‐GSDMB axis as a noncanonical immune effector mechanism [15]. This process establishes an alternative pyroptosis pathway that bypasses canonical caspase activation cascades.

## 3 CircRNAs

CircRNAs are covalently closed RNA molecules lacking a 5′ cap structure and a 3′ poly(A) tail [57]. Although circRNAs were identified more than 3 decades ago, they were long dismissed as anomalies of splicing. Fortunately, in the past decade, circRNAs have emerged as a distinct research field, uncovering their unique functional roles in disease regulation. Compared with their linear counterparts, circRNAs exhibit over tenfold greater diversity and remarkable stability [28], enabling them to participate in multiple stages of disease progression.

### 3.1 Metabolism of circRNAs

In this module, we provided a concise overview of circRNA biogenesis, degradation and subcellular localisation. However, the precise molecular regulation governing these fundamental processes continues to present significant knowledge gaps.

#### 3.1.1 The Biogenesis of circRNAs

**FIGURE 3:**
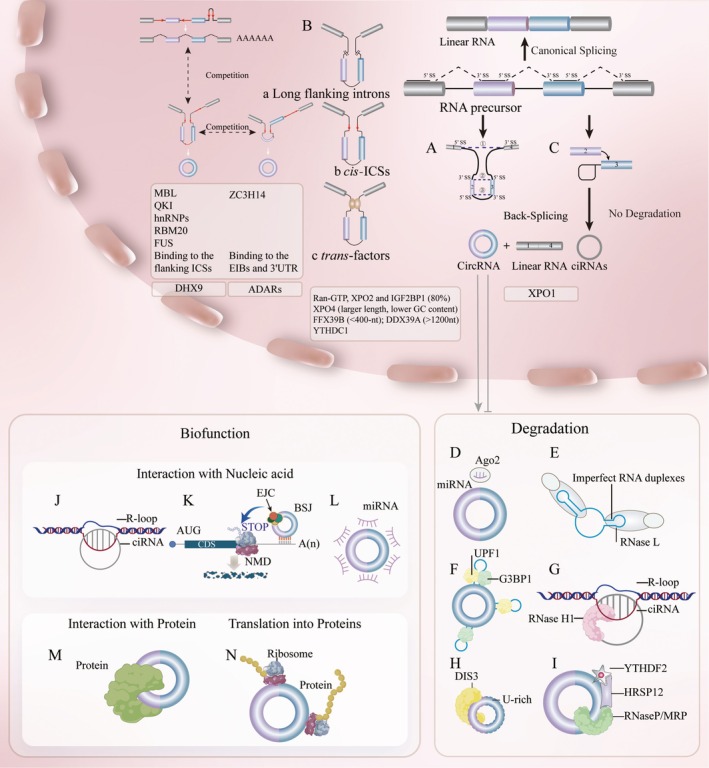
Metabolism and biofunction of circRNAs. The biogenesis of circRNAs. (A) Back‐splicing for circRNAs. Following canonical splicing ①, linear RNAs are generated alongside skipped intermediate exons. These skipped exons subsequently undergo covalent circularisation through back‐splicing ②. The final processing by canonical splicing ③ leads to the formation of circRNAs. (B) The regulators of back‐splicing. (a) long flanking introns, (b) cis‐elements/ICSs and (c) transfactors. MBL, QKI, hnRNPs, RBM20 and FUS are the RBPs that facilitate circRNAs biogenesis by binding to flanking ICSs. ZC3H14 is the RBP that facilitates circRNAs biogenesis by binding to the EIBs and 3'UTR. DHX9 suppresses circRNAs biogenesis through specific binding to inverted‐repeat Alu elements, thereby unwinding paired ICS structures. ADARs modulate circRNAs biogenesis through A‐to‐I RNA editing of flanking intronic sequences. (C) The biogenesis of ciRNAs. The degradation of circRNAs. (D) MiRNAs mediate the degradation of circRNAs through an Ago2‐dependent mechanism. (E) RNase L mediates circRNA degradation by recognising imperfect RNA duplexes. (F) UPF1 and G3BP1 mediate degradation of circRNAs with extensive dsRNA structures. (G) RNase H1 degrades ciRNAs within R‐loops through endonucleolytic cleavage of RNA–DNA hybrids. (H) DIS3 selectively degrades U‐rich circRNAs via its endoribonuclease activity. (I) The RNase P/MRP complex mediates endogenous cleavage of m6A‐modified circRNAs. The biofunction of circRNAs. (J) Interaction between ciRNAs and DNAs leads to the formation of R‐loops. (K) Interaction between circRNAs and mRNAs triggers circNMD. (L) MiRNA sponge/ceRNA mechanism. In most cases, circRNAs function as ceRNAs by sponging miRNAs, thereby alleviating miRNA‐mediated suppression of target mRNAs. (M) Interaction between circRNAs and proteins. (N) Translation of circRNAs. Created in Adobe Illustrator 2025.

Canonical alternative splicing that joins an upstream 5′ splice site (splice donor) to a downstream 3′ splice site (splice acceptor) [58]. In addition to relying on canonical alternative splicing, the generation of most circRNAs also relies on a specific splicing mode, back‐splicing, whereby the downstream 5′ splice site is ligated to the upstream 3′ splice site (Figure 3A) [21]. Both splicing processes are processed by RNA Pol II transcription and the back‐splicing is mainly coupled with transcription [21]. Inevitably, an interplay or competition is present between canonical pre‐mRNA processing and back‐splicing processing [59, 60], for example, slow RNA Pol II favours the production of linear RNAs, whereas fast RNA Pol II produces more circular forms of the same gene [59]. Moreover, transfactors that regulate pre‐mRNA alternative splicing can similarly regulate the formation of circRNAs [61]. Crosstalk exists between canonical alternative splicing and back‐splicing, but what features determine which splicing occurs remains a mystery.

Currently, the understanding of most circRNAs' biogenesis regulation is limited to long flanking introns, cis‐elements (intronic complementary sequences, ICSs) and transfactors (Figure 3B) [21]. A hypothesis suggests that back‐splicing is unfavourable for the assembly of the spliceosome compared to conventional splicing, and therefore cis‐elements and transfactors are required to stabilise the assembly of spliceosomes [62]. The presence of long flanking introns constitutes an intrinsic determinant of circRNA biogenesis. In mammalian systems, this process is usually driven by base pairing between orientation–opposite ICSs [63]. ICSs can drive the close proximity of downstream 5′ splice sites and upstream 3′ splice sites [64] and these mechanisms are conserved among species [65]. Consistently, the biogenesis of circRNAs is dramatically impaired when ICSs of the endogenous gene loci on either side of the circRNAs' parent sequence are disrupted or deletion [66, 67], and the formation of new ICSs pairs led by chromosomal translocations results in the biogenesis of novel circRNAs [68, 69]. Moreover, cross‐species analyses showed that the conservation of circRNAs is correlated with the existence of ICSs in humans and mice, but not the sequence of the circRNAs themselves [65]. Among them, Alu elements contribute most to circRNAs' formation in humans [65]. Moreover, the competition among ICSs leads to increased complexity of circRNAs. The competition between flanking inverted‐repeated Alu pairs leads to the production of multiple circular transcripts from a single pre‐mRNA [64]. Intriguingly, competition also exists between flanking ICSs and individual intron ICSs, which regulate exon circularisation efficiency (Figure 3Bb) [64, 70, 71].

In addition to the direct regulation of cis‐elements, some trans‐acting factors are also regulating back‐splicing by synergising with associated intronic cis‐elements [72]. Importantly, deletion or repression of core spliceosome components resulted in upregulated circRNAs and concomitant downregulated linear transcripts [60, 73]. This is consistent with the point above that competition is present between linear and circular transcripts. In exception to the core spliceosome components, a number of RNA‐binding proteins (RBPs) were found to synergise with flanking ICSs to modulate the formation of circRNAs [59, 61, 74, 75, 76, 77, 78, 79, 80, 81, 82, 83, 84]. Most RBPs [59, 61, 74, 75, 76, 79, 80, 81, 82, 83, 84] directly interact with the parental pre‐mRNA flanking ICSs to facilitate the formation of circRNAs, the most representative being muscleblind (MBL) [59] in flies and QKI [74] in humans (Figure 3Bc). Recently, new binding sites for RBPs regulating back‐splicing have been identified. Unlike synergistic interaction with intronic cis‐elements, ZC3H14 promotes circRNAs biogenesis by binding to the exon–intron boundaries (EIBs) of circularised exons and 3′‐untranslated region (UTR) of cognate mRNAs [85]. Among them, two studies [80, 82] indicated that Distal‐Alu‐Long‐Intron circRNAs [82] were more susceptible to being regulated by such RBPs compared to Proximal‐Alu‐Short‐Intron [82] circRNAs. Reasonably, RBPs that inhibit the formation of circRNAs are also present. DHX9, a nuclear RNA helicase, binds specifically to inverted‐repeat Alu elements leading to unwinding paired ICSs, thereby decreasing the amount of circRNAs [78, 86]. In addition to direct binding, RBPs are also able to influence circRNAs biogenesis through RNA editing. Adenosine to inosine (A‐to‐I) editing catalysed by ADARs is the most prevalent type of RNA editing. Research has shown that, ADAR1 [70, 77, 87] and/or ADAR2 [77] can promote or inhibit thousands of circRNAs via direct binding and/or editing the flanking intronic sequences, and the A‐to‐I editing can also favour the binding of RBPs thus regulating back‐splicing [77]. Co‐regulation is present based on the competitive binding capacities of RBPs to ICSs. Thus, a comprehensive understanding of collective modulation among different RBPs is warranted. Furthermore, the generation mechanisms of some specific circRNAs were preliminarily explored. The formation of circular intronic RNAs (ciRNAs) depends on a consensus motif, a 7 nt GU‐rich motif near the 5′ splice site and an 11 nt C‐rich motif close to the branchpoint site, thus escaping from debranching (Figure 3C) [88]. Moreover, a ciRNA derived from tRNAs (tricRNAs) was identified. During eukaryotic pre‐tRNA splicing, the SEN/TSEN complex uses a ‘molecular ruler’ mechanism to measure and identify the cut sites of the mature tRNA body. Subsequently, tRNA exon halves and intron ends are directly ligated by a single enzyme HSPC117/RtcB [89, 90]. Notably, although the exploration of circRNAs biogenesis is progressively deepening, the exact mechanism remains elucidated.

#### 3.1.2 The Degradation of circRNAs

The special circular structure of circRNAs confers resistance to RNA decay machineries, and because of this, even inefficient back‐splicing can enable circRNAs to accumulate to high levels [91]. Compared with homologous linear transcripts, circRNAs had higher stability, and the median half‐life of circRNAs is at least 2.5 times longer than their linear counterparts [92]. Even so, the mechanism of circRNAs' degradation has been tentatively explored.

MicroRNA (miRNA)‐mediated Argonaute 2 (Ago2)‐slicer‐dependent degradation of circRNAs was reported (Figure 3D) [93, 94]. CircRNA CDR1as was cleaved by miR‐671 via Ago2 slicer activity [93, 94]. However, until now, this type of degradation was unique. miRNA‐mediated degradation seems to rely on near‐perfect complementary pairing between circRNAs and miRNAs. Typically, circRNAs bear partially complementary sequences that act as microRNA response elements (MREs), allowing circRNAs to act as competing endogenous RNAs (ceRNAs) [26, 27, 93, 94, 95, 96].

Moreover, specific structure‐mediated degradation of circRNAs has been reported. Degradation of circRNAs containing imperfect RNA duplexes mediated by ribonuclease L (RNase L) was found to be involved in innate immune responses (Figure 3E). Previous studies reported the presence of double‐stranded RNA (dsRNA) as a marker of viral infection [97]. Upon detection of cytoplasmic dsRNA the protein kinase R (PKR) pathway and the oligo(A) synthetase(OAS)‐RNase L pathway were initiated [98]. PKR limits viral protein synthesis by binding viral dsRNA [98], while RNase L accelerates dsRNA decay to limit viral replication [98]. CircRNAs degradation has links in two pathways. The cytoplasmic endonuclease RNase L can globally degrade endogenous circRNAs, which tend to form 16–26 bp imperfect RNA duplexes and act as inhibitors of PKR, leading to PKR activation in the early stages of innate immune responses [99]. Additionally, the highly structured circRNAs (containing many dsRNA structures) decay pathway relies on the involvement of two RBPs, RNA helicase upstream frameshift 1 (UPF1) and endonuclease G3BP1. ATP‐dependent helicase UPF1 cooperates with endonuclease G3BP1 by unwinding highly structured RNA (Figure 3F). This decay pathway likewise selectively regulates the degradation of mRNAs with highly structured 3′UTR [100]. Furthermore, DNA:RNA hybrids (R‐loops) formed by ciRNA with high GC% mediate ciRNA degradation. *ci‐ankrd52* has a stronger ability of R‐loops formation compared to cognate pre‐mRNA. This leads to the release of pre‐mRNA from R‐loops, and subsequent ciRNA is degraded by RNase H1 (Figure 3G), thereby promoting efficient transcriptional elongation of ciRNA‐producing loci [101].

In addition, N6‐methyladenosine (m6A)‐modified circRNAs are associated with the degradation of circRNAs. m6A modified circRNAs are subject to endoribonucleolytic cleavage by the ribonuclease complex RNase P/MRP. The degradation is dependent on the recognition of YTHDF2 (m6A reader protein), cleavage by RNase P/MRP (endoribonucleases) and the adaptor role of HRSP12 bridging YTHDF2 and RNase P/MRP (Figure 3I) [102].

Importantly, the above‐mentioned mechanism is not specific to the degradation of circRNAs. mRNAs are degraded by miRNA‐mediated Ago2‐dependent manner [27, 95, 103]; mRNAs with highly structured 3'UTR are degraded by UPF1‐ and G3BP1‐mediated pathways [100]; RNA moiety of R‐loops is specifically degraded by RNase H1 [104, 105]; YTHDF2 destabilises m6A‐containing RNAs [106]; and viral and cellular dsRNAs cleaved by RNase L [98] have all been reported. Intriguingly, GW182 although a key component of the P‐body (involved in mRNA degradation) and RNAi machinery, mediates the degradation of circRNAs in an Ago‐slicer or P‐body independent manner [107]. However, unfortunately, the mechanism of GW182‐mediated degradation of circRNAs is not clear, and only the mid domain of GW182 contributing to circRNA degradation has been reported [107]. Recently, a novel surveillance mechanism that fine‐tunes circRNA homeostasis under physiological conditions has been characterised [108]. DIS3, through its endonuclease activity, selectively degrades endogenous U‐rich circRNAs while exhibiting minimal impact on their linear counterparts. This DIS3‐mediated circRNA decay pathway is evolutionarily conserved and operates specifically in the cytoplasmic compartment. These findings establish a rational framework for engineering therapeutic circRNAs with optimised U‐content profiles to evade endogenous degradation pathways, a strategy that may significantly enhance the pharmacokinetic properties of circRNA‐based therapeutics (Figure 3H) [108]. The maintenance of circRNA homeostasis appears to involve coordinated regulation through cell division, subcellular compartmentalisation, RNA structural conformations and functional protein assemblies. However, the molecular mechanisms integrating these processes remain elusive. The critical unresolved questions are the unidentified specialised decay machinery that selectively recognises topological features unique to circular transcripts that preserve circRNA equilibrium beyond canonical RNA surveillance systems.

#### 3.1.3 The Subcellular Localisation of circRNAs

Except circRNAs containing retained introns (ciRNAs [88] and exon–intron circRNAs (EIciRNAs) [109]), a number of circRNAs are predominantly (75%–90%) [28] localised in the cytoplasm [110], to perform biological functions [27] or undergo degradation [94, 99, 106, 107], suggesting that a pathway for exporting circRNAs from the nucleus exists.

Nuclear export of proteins and RNAs is mediated by exportins (XPOs) [111, 112], similarly, these proteins can be implicated in circRNAs nuclear export [110, 111]. The XPO family in humans consists of six members [28], among which, three are involved in the nuclear export of circRNAs [110, 111]. The assembly of circRNAs export complexes in the nucleus is dependent on Ran‐GTP, XPO2 and IGF2BP1, which export 80% of the most abundant circRNAs out of the nucleus [110]. Moreover, XPO4 is involved in the nuclear export of higher expression levels of exonic circRNAs with larger lengths and lower GC content [111]. Deficiency of intracellular XPO4 leads to the accumulation of circRNAs in the nuclear as well as the formation of R‐loops (including circRNA:DNA and linear RNA:DNA) and DNA damage [111]. While the depletion of XPO1 via CRM1 was identified to promote the nuclear export of circRNAs indirectly through upregulation of the Ran‐GTP [110].

In addition to XPOs, length‐dependent export of circRNAs was identified. The nuclear export of short (< 400 nt) and long (> 1200 nt) circRNAs is regulated by the ATP‐dependent RNA helicase DDX39B or DDX39A, respectively [113]. DDX39 is able to resolve R‐loop formation and DNA damage caused by XPO4 insufficiency [111]. Similar phenomena can be observed in Drosophila [111, 113].

Furthermore, although the export of circRNAs mediated by m6A modification has not been reported, given that the nuclear export of m6A‐modified mRNA can be mediated by the m6A‐binding protein YTHDC1 [114]. whether m6A affects the nuclear export of circRNAs deserves to be determined.

### 3.2 Mechanisms of circRNAs Biofunction

CircRNAs exert their biological functions through three primary mechanisms: (1) nucleic acid interactions mainly involving DNA [111, 115, 116], RNA [117] and miRNAs [26, 27, 93, 94, 95, 96]; (2) protein interactions with RBPs [59, 67, 99, 109, 118, 119, 120]; and (3) protein‐coding capacity [121, 122, 123, 124, 125, 126, 127, 128, 129, 130, 131, 132, 133]. Their nucleic acid interactions typically manifest through three distinct pathways: formation of R‐loops [111, 115, 116], mediation of nonsense‐mediated mRNA decay (NMD) [117] and stabilisation/degradation of miRNAs via molecular sponge effects [26, 27, 93, 94, 95, 96]. Regarding protein interactions, circRNAs regulate RBP functionality through three critical mechanisms: modulating protein activity [59, 67, 99], influencing subcellular localisation of target proteins [118, 119] and regulating the interaction between proteins [109, 120]. Notably, proteins encoded by circRNAs differ from those produced by their parental genes. The discovery of these cryptic peptides holds significant implications for advancing our understanding of disease pathogenesis [121, 122, 123, 124, 125, 126, 127, 128, 129, 130, 131, 132, 133] and developing novel vaccine strategies [57, 134]. It is noteworthy that mechanistic investigations of circRNAs continue to rely heavily on frameworks established for lncRNAs [135]. While the topological uniqueness of circRNAs arising from back‐splice junctions suggests potential functional distinctiveness, rigorous experimental validation of such structure–function relationships remains notably lacking in existing studies.

#### 3.2.1 Interaction With Nucleic Acid

CircRNA‐nucleic acid interactions encompass three primary mechanisms: (1) formation of R‐loops with genomic DNA [111, 115, 116], (2) regulation of mRNA stability via NMD [117], and (3) most notably, the molecular sponge effect mediating miRNA degradation or stabilisation through MREs [26, 27, 93, 94, 95, 96].

RNA–DNA hybrids serve as crucial intermediates throughout the mammalian genome and are involved in cellular processes such as DNA replication and transcription. Among these, R‐Loops, consisting of DNA–RNA hybrids, are formed when RNA base pairs with nontemplate DNA strands, thus displacing a single‐stranded DNA. Typically, circRNAs accumulate in the cytoplasm; however, specific circRNAs are also present in the nucleus. Some of these circRNAs could form circRNA:DNA hybrids (circR‐loops) at their cognate loci (Figure 3J) [111, 115, 116]. For instance, circSMARCA5 in breast cancer can bind exon DNA directly at its parent gene locus, thus resulting in transcriptional pausing of its parent mRNA through circR‐loops formation, as well as improved sensitivity to cytotoxic drugs [116]. Recently, Conn et al. identified circR‐loops pervasively present in chromosomal translocations between the mixed lineage leukaemia gene and translocation partners. These circR‐loops could lead to transcriptional pausing, proteasome inhibition, chromatin reorganisation, as well as DNA breakage and, importantly, drive oncogenic gene fusions through endogenous RNA‐directed DNA damage [115]. Furthermore, excessive nuclear accumulation of circRNAs would equally trigger the formation of circR‐loops and induce DNA damage [111].

The interactions between circRNA‐RNA also have an influence on the stability of mRNAs. Gene expression fidelity is participated in the maintenance of cellular homeostasis, in which sufficient quality nature mRNAs play an important role [136]. To accomplish this, surveillance systems that scrutinise the quality of mRNAs are necessary. In eukaryotic cells, the NMD is the best‐characterised mRNA surveillance pathway [136]. Recently, circRNAs were reported to be involved in NMD, which was described as circRNA‐induced NMD (circNMD) [117]. During circNMD, exon–junction complexes (EJCs) deposited onto circRNAs by back‐splicing were brought close to the 3′UTR of target mRNAs, triggering EJC‐dependent NMD, suggesting that circRNAs have therapeutic potential due to selective and prolonged downregulation capable of destabilised mRNAs (Figure 3K) [117].

Remarkably, a landmark study identified that circRNAs function as miRNAs sponges or ceRNAs through MREs, that bind miRNAs and thus prevent them from inhibiting target mRNAs (Figure 3L) [26, 27]. Hierarchical affinity of miRNA targets and miRNA and mRNA/ncRNA target pool ratios are key characteristics of circRNAs exerting ceRNA roles [103]. Testis‐specific Sry circRNA contains 16 target sites for miR‐138, thereby suppressing miR‐138 expression and is associated with testis development [27]. In vascular smooth muscle cells (VSMCs), the conserved miR‐183‐5p binding site in humans and mice enables hsa_circ_0001402 to suppress miR‐183‐5p expression and thereby regulate neointimal hyperplasia [95]. However, undiscovered mechanisms are present based on the observed phenotype [96]. Of the reported circRNAs in this category, the most striking miRNA sponge is Cdr1as (also known as ciRS‐7) [94]. Cdr1as is one of the most common target transcripts for miRNA, especially miR‐7 and miR‐671 [26, 94, 96]. Overexpression of Cdr1as/ciRS‐7 sequences produces phenotypes similar to miR‐7 knock down [26] and a positive correlation is present between mRNA with miR‐7 binding sites and Cdr1as in human cell lines [26, 93], suggesting that Cdr1as can act as a miR‐7 sponge. Similarly, Cdr1as can sponge miR‐671 [96]. However, opposite effects on miR‐7 and miR‐671 were presented in Cdr1as knockout mice. The reason may lie in the difference in the miR‐7 and miR‐671 binding sites on Cdr1as. Extensive complementarity beyond the seed region is absent in > 70 miR‐7 binding sites among Cdr1as. In contrast, only one but almost perfect complementarity of the binding site of miR‐671 is present on Cdr1as, which may contribute to the degradation of miR‐671 [96]. Whereas the presence of more than 70 miR‐7 binding sites may protect miR‐7 from target‐directed microRNA degradation (TDMD) mediated by remaining targets [137]. Subsequently, as miR‐7 transporter Cdr1as is cleaved in some cases, triggering the release of miR‐7 [26]. However, a definite conclusion that would explain this phenotype has not yet been confirmed. Therefore, further explorations on the mechanism of interactions between circRNAs and miRNAs are necessary.

#### 3.2.2 Interaction With Proteins

The formation of circRNA–protein complexes is also an important way for circRNAs to function (Figure 3M). The effects of circRNAs on proteins are summarised in the following patterns: (1) Inhibits [59, 67, 99] or promotes [138] the function of proteins. (2) Influencing the nuclear/cytoplasmic distribution of proteins [118, 119]. (3) Cementing or dissociating the interactions between proteins [109, 120].

Promotional and inhibitory effects are implicated in the regulatory role of circRNAs on protein. The classical example of inhibition of protein function by circRNAs is the interplay between Drosophila circMbl and the MBL encoded by the cognate gene [59]. MBL can specifically bind the flanking introns of circMbl and promote its production. Subsequently, interaction between mature circMbl transcripts and MBL leads to sequestered MBL. Excessive MBL leads to increased circMbl production accompanied by decreased linear MBL mRNAs and MBL protein. An efficient negative feedback loop and competition between back‐splicing and linear splicing were identified [59]. Intriguingly, dsRNA‐containing circRNA was reported to act as inhibitors of double‐stranded nucleic acid‐binding protein [67, 99]. Take Cyclic GMP‐AMP (cGAMP) synthase (cGAS) as an example. cGAS was reported as a cytosolic DNA sensor. Once binding DNA, the synthesis of cGAMP is catalysed, which ultimately leads to the production of type I interferons. The circRNA produced in D430042O09Rik is highly expressed in the nucleus of long‐term haematopoietic stem cells and acts as an antagonist for cGAS (cia‐cGAS) [67]. Mechanistically, dsRNA‐containing circRNA binds cGAS and prevents the combination between cGAS and endogenous DNA. It was proposed that double‐stranded DNA as well as DNA–RNA hybrids but not double‐stranded RNAs could activate cGAS in a sequence‐independent manner. The combination of cia‐cGAS to cGAS blocks its enzymatic activity, thus preventing cGAS from recognising self‐DNA, leading to the suppressed production of Type I interferons [67]. Similarly, circRNAs that tend to form 16–26 bp imperfect RNA duplexes could act as protein kinase R (PKR) inhibitors involved in innate immunity (see above for details) [99]. The reports on the promotion of protein function are likewise present. CircHIPK3, which was highly expressed in vulnerable plaques and H_2_O_2_‐stimulated smooth muscle cells, directly targeted mitochondrial dynamin DRP1 to enhance DRP1 activity, triggering intensified mitochondrial fragmentation and ROS production, ultimately resulting in necroptosis of VSMCs and the formation of vulnerable plaques [138].

In addition to affecting the function of proteins, circRNAs are also able to impact the nuclear/cytoplasmic distribution of proteins. Nuclear export of some proteins is facilitated in the presence of specific circRNAs. Zhang et al. reported that circLIFR‐007 acts as a negative controller of BC liver metastasis. Mechanistically, circLIFR‐007 upregulates the phosphorylation level of YAP by promoting hnRNPA1 nuclear export thereby facilitating the combination of hnRNPA1 and YAP in the cytoplasm. ultimately resulting in the inhibited transcription of specific liver transfer proteins [119]. Similarly, circRNAs likewise promote nuclear accumulation of proteins. Super‐enhancers‐related circRNA circPVT1 could recruit YBX1 into the nucleus, thus triggering RRM2 transcription. Mechanistically, the cold shock protein domain (CSD) of YBX1 plays a critical role in this process. The loss of the CSD domain reduced the affinity between YBX1 and circPVT1. Furthermore, inhibited RRM2 mediated by circPVT1 suppression could be rescued by wild‐type YBX1 but not CSD domain removed YBX1 mutants [118].

In physiological or pathological conditions, proteins chelate each other to form complexes. The circRNAs can cement or dissociate the interactions between them. The function of circRNAs that cement protein–protein interactions is often described as protein scaffolds [58, 139, 140]. Among them, the most classic example is EIciRNAs, which can enhance the interactions between Pol II and U1 snRNP at the promoters of parental genes, leading to enhanced gene expression [109]. Logically, the example that dissociates the interaction between proteins is reported [120]. Physiologically, Ccnb1 and Cdk1 form a compact complex, which allows Ccnb1 to function, leading to cell mitosis. However, in the presence of circ‐Ccnb1, the interaction between Ccnb1 and Cdk1 was dissociated, accompanied by the formation of a large complex containing circ‐Ccnb1, Ccnb1 and Cdk1, resulting in inhibited Ccnb1 function as well as suppression of tumour growth [119].

These effects encompass the ways in which circRNAs affect the downstream of the regulated proteins, thus regulating DNA modification [141], transcriptional regulation [109, 118], as well as posttranscriptional modifications [119], and so on.

#### 3.2.3 Translation Into Proteins

Translation of coded information is dependent on ribosome recruitment [142]. The 5′‐cap and 3′‐poly(A) tail play a critical role in the recruitment of ribosomes to mRNA, which is widely accepted in canonical cap‐dependent translation [142]. Due to lack of 5′‐cap and 3′‐poly(A) tail, circRNAs were seen to be devoid of coding function until engineered circRNAs with an internal ribosome entry site (IRES) made possible the cap‐independent translation of circRNAs [143]. Until now evidence indicates that the cap‐independent translation mechanisms of circRNAs include IRES [126, 127, 128], m6A [129, 130, 131], A‐to‐I editing [132] and internal initiation of translation by eIF4A3 (Figure 3N) [133].

The circRNAs translatomics characteristics exhibit significant differences compared to their parental linear mRNA‐derived proteins, primarily due to their unique looped structure and translation mechanisms. Parental linear mRNAs produce specific proteins through a single open reading frame (ORF) derived from consecutive exons, whereas circRNAs reorganise exon order via back‐splicing, generating chimeric ORFs that incorporate span back‐splice junctions (BSJs) [22] when an in‐frame stop codon appears beyond the first round of translation. For instance, the cryptic protein p‐414aa encoded by circSETD2 [14, 15] contains unique sequences distinct from the protein encoded by its parental mRNA, thereby regulating vascular remodelling [144]. The mechanisms in response to the lack of stop codons are also responsible for the differences in translation products. When the stop codons of linear mRNAs are lacking, the ribosome elongates to the poly(A) leading to the production of a nascent poly (Lysine) chain which is potentially toxic to the cells [145]. To minimise these potential toxicities, the ribosomal exit tunnel interacts with the nascent poly (Lysine) chain triggering nonstop decay, leading to rapid degradation of faulty transcripts [145]. However, if the translatable circRNAs lack stop codons and the number of nucleotides can be divided by three, multimers of proteins can be generated by an infinite open reading frame (iORF) using rolling circle translation [121, 122, 123, 124, 125]. Artificially generated circRNAs were validated earliest [125]. The endogenous nature of rolling translation has been revealed recently [121, 123]. For example, the glioblastoma natural circEGFR utilises an iORF through rolling translation to generate repeating amino acid sequences, rolling‐translated EGFR, reinforcing EGFR membrane localisation and promoting tumourigenicity in brain tumour‐initiating cells [121]. Intriguingly, rolling circle translation is not endless. The programmed −1 ribosomal frameshift mechanism terminates the rolling circle translation via mediating the generation of out‐of‐frame stop codons, suggesting the terminability of rolling circle translation [121, 146, 147].

Investigating the protein‐coding potential of circRNAs holds significant potential for elucidating cryptic peptides. However, challenges remain. Compared with canonical cap‐dependent translation, the internal translation of circRNAs exhibits lower efficiency. This inherent inefficiency renders the detection of protein and peptide products derived from circRNAs particularly challenging. Therefore, the development and application of more sensitive proteomic techniques become imperative to facilitate the identification of these cryptic peptides originating from circRNAs. The scarcity of unique epitopes within circRNA‐derived cryptic peptides poses significant challenges, as it inherently contributes to nonspecific binding and exorbitant costs in custom antibody production, both of which warrant critical consideration in experimental design. Notably, circRNAs sharing canonical IRES motifs with their linear mRNA counterparts exhibit distinct ribosome recruitment affinities. Under pathophysiological stimuli, this latent translational capacity of circRNAs is likely to undergo selective activation, where elucidating the underlying regulatory logic may unravel critical insights into disease‐specific translational control networks and their therapeutic exploitation [148]. Thrillingly, the unique covalently closed circular architecture of circRNAs, coupled with their inherently low immunogenicity, positions these molecules as promising candidates for next‐generation vaccine development [57, 134], particularly in overcoming current limitations in RNA stability and targeted immune activation [57, 134]. However, given the fundamental mechanistic divergence between circRNA and mRNA translation, decades of accumulated knowledge on maximising mRNA translational efficiency cannot be directly extrapolated to circRNAs. Consequently, systematic elucidation of circRNA translation mechanisms coupled with comprehensive mapping of their internal secondary structures emerges as an essential prerequisite for engineering circRNAs with enhanced translational capacity.

## 4 CircRNAs and Pyroptosis

Currently, the regulation of pyroptosis by circRNAs primarily centres on their role as miRNA sponges, which is largely attributed to the straightforward logic, well‐established techniques and potent downstream effects associated with the ceRNA mechanism. With further research, additional mechanisms are gradually being uncovered during circRNAs modulation of pyroptosis, such as circR‐loop [149], circRNA–protein interactions [150, 151, 152, 153, 154, 155, 156, 157, 158, 159] or endogenous protein translation from circRNAs [160]. Moreover, the canonical pyroptosis pathway has been the predominant focus in studies of circRNA‐mediated regulation. This is due to the frequent activation of the canonical pathway by various pathogens and a more mature research landscape in this area. Notably, emerging evidence indicates that noncanonical pathways, along with caspase‐3‐ and caspase‐8‐mediated pyroptosis, can also be regulated by circRNAs, indicating the close association between circRNAs and pyroptosis.

### 4.1 Canonical Pathway

Current research on the regulatory roles of circRNAs in pyroptosis predominantly focuses on canonical pathways, primarily concentrated in arterial system pathologies [149, 151, 152, 161, 162, 163, 164, 165, 166, 167, 168, 169, 170], diabetes complications [154, 155, 156, 171, 172, 173, 174, 175, 176], as well as inflammatory and infectious disorders [160, 177, 178, 179, 180, 181, 182, 183, 184, 185, 186].

#### 4.1.1 Arterial System Pathologies

Inflammatory responses play a crucial role in atherosclerosis [187], ischemia‐induced myocardial injury [188] and myocardial ischemia/reperfusion (I/R) injury [189], all of which are key pathological processes in acute myocardial infarction. Emerging research highlights the regulatory role of circRNAs in these processes through their modulation of pyroptosis [162, 163, 164, 165, 166, 167]. Evidence identifies circ_0090231 [162] and circ_0029589 [163] as key regulators of atherosclerotic progression through their distinct modulation of pyroptosis in human aortic endothelial cells (HAECs) and macrophages, respectively. Oxidised low‐density lipoprotein (ox‐LDL) treatment significantly upregulates circ_0090231 [162] expression in HAECs. This circRNA exacerbates atherosclerotic progression by functioning as a ceRNA for miR‐635, thereby elevating expression of NLRP3, the canonical inflammasome component targeted by miR‐635, which subsequently aggravate pyroptosis. Guo et al. identified circ_0029589 [163] as a downstream effector of interferon regulatory factor‐1 (IRF‐1) in modulating macrophage pyroptosis. Mechanistically, IRF‐1 suppresses hsa_circ_0029589 expression by promoting m6A modification and upregulating methyltransferase‐like 3 (METTL3) in macrophages, thereby triggering pyroptosis. Moreover, in ischemic myocardium of MI mice and hypoxia‐exposed neonatal mouse ventricular cardiomyocytes (NMVCs), circHelz was demonstrated significant upregulation [164]. Mechanistically, circHelz exacerbates myocardial injury through sponge miR‐133a‐3p, thereby activating the NLRP3 inflammasome‐mediated proinflammatory cascade and subsequent cardiomyocyte pyroptosis. Knockdown of circHelz post‐MI markedly attenuated pyroptosis and reduced infarct size, confirming its pathogenic role in postischemic cardiac remodelling. Furthermore, emerging evidence further elucidates the role of circRNAs in myocardial I/R injury through their regulation of pyroptosis in cardiomyocytes [165, 166, 167]. In both cultured cardiomyocytes subjected to anoxia/reoxygenation (A/R) and mice cardiac tissues following I/R injury, circ‐NNT demonstrated significant upregulation. Mechanistically, circ‐NNT exacerbates cardiomyocyte pyroptosis by functioning as ceRNA for miR‐33a‐5p, thereby upregulated ubiquitin‐specific protease 46 (USP46). This regulatory axis culminates in activation of pyroptosis‐executing caspases (caspase‐1 and caspase‐11) and elevated levels of proinflammatory cytokines (IL‐1β and IL‐18), ultimately driving pyroptosis in cardiomyocytes [165]. Similarly, circHMGA2 [166] exhibited significant upregulation in both A/R‐treated human cardiomyocytes (HCMs) and myocardial tissues from mice subjected to I/R injury. Notably, circHMGA2 demonstrated functional capacity to exacerbate A/R‐induced pyroptosis in HCMs. Additionally, studies implicate circPAN3 in mediating the cardioprotective effects of sevoflurane against myocardial ischemia–reperfusion injury (MIRI) [167]. Preconditioning with sevoflurane effectively counteracts the A/R‐induced downregulation of circPAN3 expression in HCMs. Mechanistic investigations reveal that circPAN3 exerts its protective role by negatively regulating miR‐29b‐3p, thereby upregulating stromal cell‐derived factor 4 (SDF4). Notably, genetic silencing of circPAN3 or overexpression of miR‐29b‐3p abrogated alleviatory effects of sevoflurane in vitro cardiomyocyte injury and HCMs pyroptosis. By orchestrating inflammatory cell death pathways in cardiomyocytes, endothelial cells and macrophages, these circRNAs influence the pathophysiological continuum of MI. The cell‐type‐specific regulatory patterns of circRNAs in controlling inflammatory cascades highlight their potential for developing precision diagnostics and targeted antipyroptotic therapies to mitigate MI‐associated tissue damage and adverse remodelling.

Neuroinflammatory responses play a pivotal role in ischemic stroke [190, 191]. Emerging evidence implicates pyroptosis in the pathogenesis cascade. This pyroptosis‐mediated signalling cascade establishes a pathological feedforward loop, amplifying neuroinflammatory processes that culminate in progressive parenchymal destruction within the cerebral microenvironment [192]. Recent investigations have revealed that circRNAs participate in ischemic stroke progression through their regulatory effects on pyroptosis. Sun et al. reported that in vitro modelling of cerebral ischemia using oxygen–glucose deprivation (OGD)‐treated primary hippocampal neurons revealed a pathological elevation of circ_NLRP1 [168]. This circRNA was found to trigger NLRP3 inflammasome‐mediated pyroptosis via sponging mmu‐miR‐199b‐3p. Notably, mmu_circ_0001113 (circFndc3b) exhibited significant downregulation in the penumbral cortex of middle cerebral artery occlusion mouse models [151]. Exercise intervention upregulated circFndc3b expression in microglia/macrophages, correlating with reduced pyroptosis, attenuated infarct volume and enhanced neurological recovery. Mechanistically, the interaction between circFndc3b and Enolase 1 (ENO1) facilitates ENO1 binding to the 3'UTR of Krüppel‐like factor 2 (Klf2) mRNA, thereby stabilising Klf2 transcripts and elevating its protein expression [151]. This molecular cascade suppresses NLRP3 inflammasome‐mediated pyroptosis in microglia/macrophages [151]. Furthermore, the circFndc3b‐ENO1 complex promotes ENO1 interaction with the 3′UTR of Fused in Sarcoma (FUS) mRNA, resulting in increased FUS protein levels that reinforce circFndc3b cyclisation, establishing a positive feedback loop [151]. These prototypical investigations elucidating circRNA‐mediated regulation of neuronal survival mechanisms under ischemic stress establish circRNAs as promising candidates for developing multitarget therapeutic strategies in ischemic stroke. The dual regulatory paradigms demonstrated by circ_NLRP1 (miRNA sponging) and circFndc3b (RNA‐binding protein scaffolding) particularly highlight their potential as both diagnostic biomarkers and pathomechanistic modulators for cerebrovascular therapeutics.

Furthermore, circRNAs have also been reported to modulate pyroptosis in the pathological progression of additional ischemic pathologies and ischemia–reperfusion injury‐associated diseases. CircHIPK3 has been implicated in the therapeutic effects of exosomes derived from human umbilical cord mesenchymal stem cells (UMSC‐Exos) on skeletal muscle ischemic injury [169]. In mice models of skeletal muscle ischemia, circHIPK3 expression was markedly downregulated, whereas UMSC‐Exos treatment significantly restored circHIPK3 levels, concomitant with improved hemodynamic perfusion, enhanced ambulatory capacity and increased muscle strength [169]. Mechanistically, UMSC‐Exos administration suppressed NLRP3 inflammasome activation and subsequent pyroptosis in ischemic tissues, an effect substantially reversed by si‐circHIPK3. Further investigations identified the miR‐421/FOXO3a axis as a downstream target of circHIPK3, with experimental validation confirming this regulatory interaction in C2C12 cells [169]. Moreover, hepatic ischemia–reperfusion injury (IRI), a common complication in liver surgery and transplantation, involves multifaceted pathological processes including oxidative stress and inflammatory cascades [161]. Notably, circRNA‐Phf21a_0002 expression was found to be significantly downregulated in hepatic IRI. Intriguingly, this downregulation may represent an intrinsic protective mechanism, as overexpression of circRNA‐Phf21a_0002 exacerbated NLRP3 inflammasome‐mediated pyroptosis in AML12 hepatocytes subjected to I/R challenge [161]. Mechanistic interrogation revealed that circRNA‐Phf21a_0002 exerts its regulatory effects through sponging let‐7b‐5p, thereby mediating pyroptosis in AML12 cells and potentiating hepatic IRI pathology [161].

Pyroptosis is implicated in pulmonary vascular remodelling [193], the key pathology of pulmonary hypertension (PH) [194]. Notably, circRNAs have been identified as regulatory mediators in this process, with emerging studies suggesting their role in modulating pyroptosis‐associated inflammatory cascades. CircSSR1 exhibited significant downregulation in hypoxic cardiomyocytes [152]. Its overexpression demonstrated efficacy in suppressing hypoxia‐induced pyroptosis in pulmonary arterial smooth muscle cells (PASMCs) across both in vivo and in vitro models [152]. Mechanistically, circSSR1 binds to YTH domain‐containing family protein 1 (YTHDF1) to promote m6A‐dependent translational enhancement of SSR1 protein, thereby activating endoplasmic reticulum stress pathways that drive pyroptosis in PASMCs [152]. Notably, circ‐Calm4 [170] and circLrch3 [149] were identified as markedly upregulated in PASMCs, with genetic knockdown of either significantly attenuating hypoxia‐induced pyroptotic phenotypes. While both circRNAs contribute to pyroptosis, their mechanistic pathways were distinct. CircLrch3 [149] facilitates pyroptosis through R‐loop formation with its host gene Lrch3, thereby inducing coordinated upregulation of both Lrch3 mRNA and protein expression. In a distinct regulatory paradigm, circ‐Calm4 [170] exacerbates pyroptotic signalling via a ceRNA mechanism by sponging miR‐124‐3p, which leads to derepression of miR‐124‐3p target gene programmed cell death protein 6 (PDCD6) and subsequent amplification of hypoxia‐driven pyroptosis. The distinct mechanistic profiles of circSSR1 [152], circ‐Calm4 [170] and circLrch3 [149] in PH converge on pyroptosis amplification through three distinct yet complementary pathways: circSSR1 mediates m6A‐dependent translational control of endoplasmic reticulum stress effectors [152], circLrch3 facilitates R‐loop‐driven transcriptional activation of its host gene Lrch3 [149], and circ‐Calm4 operates via a ceRNA axis targeting miR‐124‐3p/Pdcd6 [170]. These mechanistically segregated epitranscriptomic regulations synergistically drive NLRP3 inflammasome hyperactivity, positioning circRNAs as nodal regulators of inflammatory vascular remodelling.

The inflammatory process is pivotal to both the initiation and progression of aneurysmal disease, persisting throughout the entire continuum of abdominal aortic aneurysm (AAA) pathogenesis [195]. Evidence has implicated circRNAs‐regulated pyroptosis in the molecular mechanisms driving AAA development and progression. Cai et al. demonstrated a marked elevation of circHipk3 expression in AAA [153]. Experimental overexpression of this circRNA‐induced macrophage pyroptosis, subsequently promoting the synergistic effect of inflammatory mediators and matrix metalloproteinase activity within the AAA microenvironment. This molecular mechanism was shown to significantly potentiate aneurysm progression in both angiotensin II (Ang II)‐ and porcine pancreatic elastase‐induced mice AAA models [153]. Chromatin Isolation by RNA Purification (ChIRP) analysis revealed that circHipk3 exerts its propyroptotic effects through dual molecular pathways [153, 196]. Mechanistic studies demonstrated direct interaction between circHipk3 and STAT3, resulting in enhanced NLRP3 expression within aortic tissues [153]. Concurrently, the circRNA facilitates Snd1‐mediated degradation of Ptbp1 mRNA through binding Snd1, thereby suppressing autophagy [153]. This coordinated dysregulation of inflammasome activation and autophagy inhibition was shown to critically mediate macrophage pyroptosis in aneurysm pathogenesis.

#### 4.1.2 Diabetes Complications

The macrovascular and microvascular complications (DXD, diabetic kidney disease; DCM, diabetic cardiomyopathy; DR, diabetic retinopathy) constitute the primary determinants of morbidity and mortality in patients with diabetes [197]. Emerging evidence implicates a pathological cascade initiated by intracellular hyperglycaemia. This metabolic derangement drives excessive reactive oxygen species (ROS) generation, ultimately establishing a sustained proinflammatory milieu, which drives diabetic complications [198].

DKD is the leading cause of end‐stage renal disease globally [199], which has been mechanistically linked to pyroptosis‐propelled inflammatory cascades [200]. Mechanistic studies have elucidated that circRNAs serve as modulators in this process. Three circRNAs—circ_0004951 [171], circCOL1A2 [172] and circ_0000181 [173]—exhibit conserved upregulation in hyperglycaemic renal environments: circ_0004951 [171] and circCOL1A2 [172] are significantly elevated in DKD patients and HG‐treated HK‐2 cells and circ_0000181 [173] is upregulated in diabetic mouse renal tubular epithelial cells (MRTECs). Notably, all three circRNAs mechanistically promote pyroptosis through sponging miRNA, selectively sequestering miR‐93‐5p (targeting NLRP3) [171], miR‐424‐5p (regulating SGK1) [172] and miR‐667‐5p (modulating NLRC4) [173], thereby accelerating DKD progression. Evidence has also documented the regulatory roles of circRNAs in pyroptosis modulation within glomerular endothelial cells (GEnCs) [174]. Under HG conditions, circ8411 demonstrates significant downregulation in GEnCs [174]. Mechanistically, this circRNA exerts its biological function through direct binding to the 3′‐UTR of miR‐23a‐5p. This molecular interaction culminates in the upregulation of ATP‐binding cassette transporter A1 (ABCA1), which subsequently mitigates intracellular cholesterol accumulation and attenuates pyroptotic cell death [174].

DCM, a metabolic cardiac disorder driven by persistent hyperglycaemia and concomitant metabolic/endocrine dysregulation, develops through multifaceted pathogenic mechanisms culminating in myocardial injury [201]. Substantial experimental evidence establishes that pyroptosis‐triggered inflammatory cascades involved in this disease progression [202], which is also dynamically modulated by circRNAs. The circRNA hsa_circ_0131202 (DICAR) was significantly downregulated in cardiac tissues of diabetic mice and demonstrated cardioprotective effects against DCM [154]. Genetic manipulation studies revealed that heterozygous DICAR knockout (*DICAR* ^*+/−*^) mice developed spontaneous cardiac dysfunction accompanied by characteristic pathological features including cardiomyocyte hypertrophy and myocardial fibrosis [154]. Conversely, cardiac‐specific overexpression of DICAR in transgenic mice (*DICAR* ^*Tg*^) markedly attenuated diabetes‐induced cardiomyopathy progression [154]. At the cellular level, experiments demonstrated that DICAR overexpression effectively suppressed pyroptosis in advanced glycation end products (AGEs)‐stimulated cardiomyocytes, whereas DICAR knockdown exacerbated this PCD pathway. Mechanistically, DICAR exerts its antipyroptotic effects through direct interaction with valosin‐containing protein (VCP). This molecular interaction stabilises Med12 protein by preventing VCP‐mediated protein degradation, thereby disrupting the pyroptotic signalling cascade [154]. Furthermore, hsa_circ_0076631 expression demonstrated significant upregulation in both hyperglycaemic cardiomyocytes and diabetic patient serum samples. Mechanistic analysis revealed this circRNA aggravate pyroptosis through modulation of the miR‐214‐3p/caspase‐1 signalling axis [175].

Studies have demonstrated that pyroptosis‐mediated cellular death and dysfunction play a pivotal role in the progression of diabetic retinopathy [203], a process modulated by circRNAs. CircFAT1 demonstrated marked downregulation in both proliferative fibrovascular membranes from DR patients and HG‐stimulated retinal pigment epithelial (RPE) cells [155]. Mechanistic investigations revealed that in HG‐exposed RPE cells, circFAT1 interacts with the m6A reader protein YTHDF2, thereby inducing autophagic activation through LC3B upregulation while concurrently suppressing pyroptosis via GSDMD downregulation [155]. Furthermore, in human ARPE‐19 RPE cells exposed to DR‐related HG conditions, circZNF532 was significantly upregulated and functionally demonstrated to attenuate HG‐induced RPE apoptosis and pyroptosis through modulation of the miR‐20b‐5p/STAT3 signalling axis [176].

Comorbidity of Type 2 diabetes mellitus (T2DM) and nonalcoholic fatty liver disease (NAFLD) induces exacerbated oxidative stress, thereby triggering chronic inflammation [204, 205]. Umbilical cord‐derived mesenchymal stem cells (UCMSCs) exhibit therapeutic potential for diabetes mellitus with nonalcoholic fatty liver disease (DM‐NAFLD) by ameliorating hyperglycaemia, attenuating lipid accumulation and mitigating hepatic steatosis [206]. These therapeutic effects are primarily mediated through their secreted extracellular vesicles (EVs). Mechanistic studies further suggest that circRNA‐regulated pyroptosis may serve as a modulator within this EV‐driven therapeutic axis. In DM‐NAFLD models, circ‐Tulp4 expression was significantly reduced [156]. Administration of UCMSCs‐EVs effectively delivered circ‐Tulp4 to hepatocytes, rescuing its expression. Mechanistically, the circ‐Tulp4 formed a functional complex with lysine demethylase 6B (KDM6B), mediating specific demethylation of H3K27me3 [156]. This epigenetic remodelling enhanced H3K27me3 recruitment to the heterogeneous nuclear ribonucleoprotein C (HNRNPC) promoter, leading to transcriptional repression of HNRNPC and downregulation of its downstream target ABHD6 [156]. Importantly, overexpression of either HNRNPC or ABHD6 abolished the therapeutic effects of UCMSCs‐EVs, exacerbating pyroptosis and hepatic steatosis in DM‐NAFLD progression [156].

Emerging evidence establishes circRNAs as key regulators of pyroptosis in diabetic complications, primarily through miRNA sponge and interaction with protein scaffolding. These findings unveil therapeutic opportunities through circRNA‐based interventions: Stabilising protective circRNAs or engineering synthetic mimics could mitigate pyroptotic damage, while selectively targeting pathogenic circRNA networks may synergise with conventional antidiabetic therapies to combat complication progression.

#### 4.1.3 Inflammatory and Infectious Disorders

Pyroptosis, a lytic programmed cell death pathway characterised by inflammatory cytokine release, significantly contributes to the pathogenesis of inflammatory and infectious disorders through its dysregulated activation. Notably, pharmacological inhibition of GSDMD shows considerable therapeutic potential in the management of sepsis [207, 208]. Emerging evidence underscores the pivotal role of circRNA‐mediated pyroptosis in diverse inflammatory and infectious pathologies.

Compelling evidence identifies pyroptosis as a pivotal mediator in sepsis, with therapeutic modulation of this inflammatory cell death pathway demonstrating efficacy in alleviating sepsis‐induced pathological injury [209]. Emerging mechanistic studies further implicate circRNAs in orchestrating this pathological cascade through pyroptotic pathways. CircMLH3 was significantly upregulated in whole blood samples from septic patients and showed a strong association with clinical survival rates [181]. Functional studies demonstrated that circMLH3 overexpression promotes macrophage pyroptosis by acting as a ce RNA for miR‐590‐3p, leading to TAK1 upregulation [181]. This mechanism sequentially triggers NLRP3 inflammasome activation, caspase‐1 cleavage and ultimately exacerbates macrophage pyroptosis [181]. Moreover, circ_0075723 was significantly downregulated in patients with pneumonia‐induced sepsis [180]. Mechanistic investigations revealed that circ_0075723 suppresses macrophage pyroptosis by sponging miR‐155‐5p, thereby upregulating SHIP1 expression [180]. Emerging evidence further implicates circRNAs‐regulated pyroptosis in sepsis‐associated organ injury. In hepatic tissues of cecal ligation and puncture‐induced septic mice, the expression of mmu_circ_0001432 (circ‐Katnal1) was markedly upregulated [179]. Genetic silencing of circ‐Katnal1 significantly suppressed LPS‐induced pyroptosis in RAW264.7 monocytic macrophages [179]. Mechanistic dissection revealed that circ‐Katnal1 exacerbates hepatic injury through a miR‐31‐5p/GSDMD axis, thereby amplifying pyroptosis‐dependent parenchymal damage in septic livers [179]. Furthermore, in both *Candida albicans* ‐ and LPS‐induced septic acute kidney injury (SAKI) models (murine and cellular), circHIPK3 was observed to be significantly upregulated [178]. Mechanistic investigations revealed that circHIPK3 exacerbates SAKI‐associated inflammation through a ceRNA mechanism [178]. Specifically, circHIPK3 sponges miR‐124‐3p, thereby relieving miR‐124‐3p‐mediated suppression of KLF6 expression. The consequent upregulation of KLF6 facilitates its interaction with NLRP3, which potentiates NLRP3/caspase‐1‐dependent pyroptotic signalling. This cascade ultimately amplifies the inflammatory cascade in SAKI pathogenesis [178]. As pyroptosis emerges as a central mechanism in sepsis pathogenesis, GSDMD‐targeting therapies have entered clinical testing. The newly characterised regulatory role of circRNAs in pyroptotic pathways positions these circular transcripts as promising therapeutic candidates, either as primary targets for precision intervention or as adjuvant modulators to complement existing anti‐inflammatory strategies in sepsis management.

Inflammatory responses constitute the pathophysiological foundation of immune‐related disorders, with pyroptosis implicated as a contributing effector mechanism in these processes [210]. Current evidence suggests that circRNAs act as regulatory factors interfacing with pyroptotic signalling through multiple molecular modalities. The accumulating evidence on hsa_circ_0044235 [182], circGMCL1 [184] and hsa_circ_0008039 (circPRKAR1B) [183] highlights their distinct therapeutic potentials in immune‐related disorders. Mechanistically, hsa_circ_0044235 [182] ameliorates rheumatoid arthritis (RA) progression through the miR‐135b‐5p/SIRT1 axis, demonstrating anti‐inflammatory and antipyroptotic effects in both collagen‐induced arthritis mouse models and fibroblast‐like synoviocytes (FLSs) [182]. Similarly, circGMCL1 [184] exhibits disease‐modifying properties in Crohn's disease (CD) by orchestrating a ceRNA network that enhances cytoprotective autophagy while suppressing NLRP3 inflammasome activation, with therapeutic efficacy validated via PLGA‐based nanodelivery systems. In contrast, the pathogenic role of hsa_circ_0008039 [183] in exacerbating colonic pyroptosis positions it as a promising inhibitory target for CD intervention. Collectively, these findings underscore the translational value of circRNA‐based strategies—either through targeted overexpression of protective circRNAs (e.g., hsa_circ_0044235 [182], circGMCL1 [184]) or silencing of deleterious variants (e.g., hsa_circ_0008039 [183])—for precision management of immune‐mediated pathologies.

Emerging evidence further implicates circRNA‐regulated pyroptosis in additional inflammatory and infectious pathologies. In allergic rhinitis (AR) pathogenesis, hsa_circ_0000520 demonstrates significant upregulation in nasal lavage fluid and inferior turbinate mucosal specimens from AR patients, as well as in serum and nasal mucosal tissues from ovalbumin‐induced AR murine models [185]. Mechanistically, this circRNA acts as a sponge for miR‐556‐5p, thereby alleviating miRNA‐mediated suppression of NLRP3 through direct targeting of its 3′‐UTR. This regulatory cascade culminates in NLRP3 upregulation and subsequent pyroptosis in AR pathophysiology [185]. Moreover, the investigations in periodontal disease reveal elevated circ_0036490 expression in both periodontal ligament tissues from periodontitis patients and human gingival fibroblasts (HGFs) [186]. This circRNA facilitates pyroptosis by sponging miR‐29a, thereby derepressing DKK1 expression [186]. Cutting‐edge investigations have elucidated the mechanistic basis of circRNA‐mediated pyroptosis regulation in traditional Chinese medicine interventions for acute pancreatitis (AP) [177]. In both murine AP models and MPC‐83 pancreatic acinar cells, circHipk3 was found to be upregulated, where it promotes NLRP3 inflammasome activation through the miR‐193a‐5p [177]. Therapeutic administration of Qingjie Huagong decoction significantly attenuated circHipk3‐mediated pyroptotic pathways, correlating with marked amelioration of AP [177]. Notably, recent studies have revealed circRNAs with protein‐coding potential during infectious processes [160]. Xu et al. identified mmu_circ_0011319 (circCDC42) as a translationally competent circRNA upregulated in alveolar macrophages (AMs) from *Klebsiella pneumoniae* (KP)‐infected mice [160]. Functional characterisation revealed its capacity to encode a 165‐amino acid cryptic polypeptide via an IRES‐mediated translation mechanism [160]. Post‐KP infection, CDC42‐165aa shows marked overexpression in both MH‐S cells and primary AMs. Structural analysis revealed sequence homology between the C‐terminal 1–161 amino acids of CDC42‐165aa and its parental mRNA‐encoded CDC42 protein, suggesting a competitive inhibitory relationship [160]. Biochemical studies demonstrated that CDC42‐165aa impairs CDC42 GTPase activation by competitively binding to the guanine nucleotide exchange factor DOCK8. KEGG pathway analysis implicated CDC42‐165aa in canonical pyroptosis signalling [160]. Subsequent mechanistic investigations established that CDC42‐165aa exacerbates KP‐induced pulmonary injury through Pyrin inflammasome activation and subsequent inflammatory cascade [160].

#### 4.1.4 Miscellaneous Roles

Accumulating data demonstrate that caspase‐1‐dependent pyroptosis mediated by circRNAs drives pathophysiological progression in diverse organ systems, including neurological [211, 212], renal [213, 214], pulmonary [215, 216, 217] and hepatic [218].

In female rat models of spinal cord injury (SCI), a significant elevation of circ0000381 expression was observed [212]. Mechanistically, this circRNA appears to function as a compensatory regulator attenuating microglial/macrophage pyroptosis following SCI, as supported by two findings: the delayed upregulation of circ0000381 compared to NLRP3 induction post‐LPS stimulation, and the potentiation of pyroptosis upon circ0000381 silencing [212]. Furthermore, therapeutic investigations revealed the critical involvement of circ_003564 in mediating the neuroprotective effects of bone marrow mesenchymal stem cells (BMSCs)–derived exosomes [211]. Administration of BMSCs exosomes markedly improved functional recovery and reduced inflammasome‐associated pyroptosis in SCI rats, effects that were substantially diminished when circ_003564 expression was genetically suppressed [211].

Emerging reports highlight the critical involvement of circRNA‐regulated pyroptosis pathways in renal pathophysiology [214]. Human urine‐derived stem cells (USCs) demonstrate therapeutic potential for acute kidney injury (AKI), particularly through their exosomal delivery system [214]. CircDENND4C was identified as the most significantly downregulated circRNA in renal tissues of I/R‐injured rats. USC‐derived exosomes (USC‐Exos) effectively restored circDENND4C levels, subsequently suppressing NLRP3 inflammasome activation via the miR‐138‐5p/FOXO3a axis, thereby attenuating pyroptosis and ameliorating AKI progression [214]. Beyond acute injury, circACTR2 has been implicated in macrophage‐renal tubular epithelial cells (TECs) crosstalk during chronic fibrotic remodelling [213]. Unilateral ureteral obstruction (UUO)‐induced renal fibrosis models revealed marked circACTR2 upregulation, which mechanistically promotes NLRP3 inflammasome assembly, pyroptosis, and inflammatory activation in macrophages through sponging miR‐561 [213]. This cascade culminates in IL‐1β hypersecretion, which induces fascin‐1‐mediated TEC fibrosis. Notably, genetic ablation of circACTR2 or overexpression of miR‐561 effectively reversed fibrotic pathology, establishing this axis as a druggable target for renal fibrosis intervention [213].

Recent reports highlight the critical involvement of circRNA‐regulated canonical pyroptosis pathways in pulmonary pathologies. In radiotherapy‐irradiated A549 lung adenocarcinoma (LUAD) cells, significant downregulation of circNEIL3 was observed, with its overexpression effectively suppressing pyroptosis [216]. Mechanistic investigations revealed that circNEIL3 exerts its antipyroptotic effect through sponging miR‐1184, which subsequently alleviates miRNA‐mediated repression of PIF1. This molecular cascade ultimately induces DNA damage and activates the AIM2 inflammasome pathway [216]. CircNEIL3 knockout demonstrated significant radiation sensitivity potential in therapeutic applications [216]. Beyond neoplastic conditions, distinct circRNA regulatory patterns were reported in silica‐induced pulmonary pathology [217]. Elevated expression of circRNA11:120406118|12,040,782 was detected in serum exosomes from silicosis patients, murine silicosis models and silica‐stimulated macrophages/fibroblasts [217]. This circRNA aggravates silica‐induced macrophage pyroptosis through the miR‐30b‐5p/NLRP3 regulatory axis [217]. Interestingly, parent gene‐circRNA regulatory dynamics have been implicated in acute lung injury (ALI) pathogenesis [215]. Phorbol 12‐myristate 13‐acetate (PMA)/LPS‐treated THP‐1 macrophages (an established ALI model) exhibited reduced circ‐CARD8 expression. Functional studies demonstrated that circ‐CARD8 serves as a ceRNA for has‐miR‐580‐3p, thereby upregulating CARD8 expression and potentiating LPS‐induced macrophage pyroptosis [215].

Expanding beyond pulmonary disorders, emerging evidence implicates circRNA‐mediated pyroptosis in the pathogenesis of NAFLD [218]. Systematic investigations revealed significant upregulation of circSOD2 in both in vivo and in vitro NAFLD models [218]. Mechanistically, miR‐532‐3p directly binds to the 3′UTR of TXNIP, suppressing the TXNIP/NLRP3 inflammasome axis and its downstream inflammatory and pyroptotic cascades [218]. Notably, circSOD2 functions as a ceRNA that inhibits miR‐532‐3p‐mediated repression of TXNIP, thereby potentiating cellular pyroptosis in hepatic cells [218]. CircRNAs associated with canonical pyroptosis regulation are summarised in Table 1, which represents the most common pathway in circRNA‐mediated regulation of pyroptosis, accounting for five‐sixths of reported cases.

**TABLE 1:** Summary of circRNAs associated with the canonical pyroptosis pathway.

Classification of diseases	Diseases/Therapies	CircRNAs	Cells	Functions	Effect on pyroptosis
Arterial system pathologies	Atherosclerosis	Circ_0090231 [162]	Ox‐LDL‐treated HAECs	miR‐635/NLRP3	Aggravate
	Acute coronary syndrome and atherosclerosis	Hsa_circ_0029589 [163]	Macrophages isolated from acute coronary syndrome patients	Caspase‐1	Inhibit
	MI	CircHelz [164]	Hypoxia‐treated NMVCs	miR‐133a‐3p/NLRP3	Aggravate
	MI	Circ‐NNT [165]	A/R‐treated cardiomyocytes	miR‐33a‐5p/USP46/caspase‐1 and caspase‐11	Aggravate
	Myocardial ischemia–reperfusion injury	CircHMGA2 [166]	Hypoxia/reoxygenation‐treated HCMs	NLRP3	Aggravate
	MIRI/Sevoflurane	CircPAN3 [167]	Hypoxia/reoxygenation‐treated HCMs	miR‐29b‐3p/SDF4/NLRP3	Inhibit
	Ischemic stroke	Circ_NLRP1 [168]	OGD‐treated primary hippocampal neuronal cells	mmu‐miR‐199b‐3p/NLRP3	Aggravate
	Ischemic stroke/Exercise	CircFndc3b/mmu_circ_0001113 [151]	Peri‐infarct cortex isolated microglia/macrophages	ENO1/Klf2/NLRP3 (circRNA–protein interactions)	Inhibit
	Ischemic injury of the skeletal muscle/human UMSC‐Exo	CircHIPK3 [169]	Murine myoblast line (C2C12) cells	miR‐421/FOXO3a/NLRP3	Inhibit
	Hepatic ischemia–reperfusion injury	CircRNA‐Phf21a_0002 [161]	Hypoxia/reoxygenation‐treated AML12 cells	let‐7b‐5p/Bach1/caspase‐1	Aggravate
	PH	CircSSR1 [152]	Hypoxia‐treated PASMCs	YTHDF1/SSR1/NLRP3 (circRNA–protein interactions)	Aggravate
	PH	Circ‐Calm4 [170]	Hypoxia‐induced PASMCs	miR‐124‐3p/PDCD6/NLRP3	Aggravate
	PH	CircLrch3 [149]	Hypoxia‐treated PASMCs	Lrch3 (R‐loop)/NLRP3	Aggravate
	AAA	CircHipk3/mmu_circ_0001052 [153]	LPS‐treated macrophage	Stat3/NLRP3; Snd1/Ptbp1 (circRNA–protein interactions)	Aggravate
Diabetes complications	DKD	Circ_0004951 [171]	HG‐treated human renal tubular epithelial cells (HK2)	miR‐93‐5p/NLRP3	Aggravate
	DKD	CircCOL1A2 [172]	HG‐treated proximal tubular epithelial cells (HK2)	miR‐424‐5p/ SGK1/NLRP3	Aggravate
	DKD	Circ_0000181 [173]	Flagellin‐treated MRTECs	miR‐667‐5p/NLRC4	Aggravate
	DKD	Circ8411 [174]	HG‐treated GEnCs	miR‐23a‐5p/ABCA1/caspase‐1	Inhibit
	DCM	Hsa_circ_0131202 [154]	AGEs‐treated cardiomyocytes	VCP/Med12/NLRP3 (circRNA–protein interactions)	Inhibit
	DCM	Hsa_circ_0076631 [175]	HG‐treated cardiomyocyte	miR‐214‐3p/NLRP3	Aggravate
	DR	CircFAT1 [155]	HG‐induced RPE cells	YTHDF2/LC3B; GSDMD (circRNA–protein interactions)	Inhibit
	DR	CircZNF532 [176]	HG‐treated RPE cells	miR‐20b‐5p/STAT3; NLRP3	Aggravate
	DM‐NAFLD/UCMSCs‐derived EVs	Circ‐Tulp4 [156]	Primary mouse hepatocytes	KDM6B/H3K27me3/HNRNPC/ABHD6/NLRP3 (circRNA–protein interactions)	Inhibit
Inflammatory and infectious disorders	Sepsis	CircMLH3 [181]	LPS‐treated macrophage	miR‐590‐3p/TAK1/caspase‐1/NLRP3	Aggravate
	Pneumonia‐induced sepsis	Circ_0075723 [180]	LPS/Nigericin activated THP‐1 cells	miR‐155‐5p/SHIP1/TLR4; NLRP3	Inhibit
	Sepsis‐induced liver injury	Circ‐Katnal1 [179]	LPS‐treated RAW264.7 cells	miR‐31‐5p/NLRP3	Aggravate
	SAKI	CircHIPK3 [178]	LPS‐treated human renal tubular epithelial cells (HK2)	miR124‐3p/KLF6/NLRP3	Aggravate
	RA	Hsa_circ_0044235 [182]	LPS/ATP‐treated FLSs	miR‐135b‐5p/SIRT1/NLRP3	Inhibit
	CD	CircPRKAR1B [183]	Primary epithelial cells	NLRP3; SPTBN1	Aggravate
	CD	CircGMCL1 [184]	Epithelial cells isolated from CD patients or NCs	miR‐124‐3p/ANXA7; NLRP3	Inhibit
	AR	Hsa_circ_0000520 [185]	LPS‐treated human nasal epithelial cells	miR‐556‐5p/NLRP3	Aggravate
	Periodontitis	Circ_0036490 [186]	LPS‐treated HGFs	miR‐29a/DKK1/caspase‐1	Aggravate
	*Klebsiella pneumoniae* ‐infected	CircCDC42 [160]	*Klebsiella pneumoniae* ‐infected alveolar macrophages	CDC42‐165aa/CDC42 GTPase/Pyrin (translate into proteins)	Aggravate
	AP/Qingjie Huagong decoction	CircHipk3 [177]	Caerulein‐treated MPC‐83 cells	miR‐193a‐5p/NLRP3	Aggravate
Miscellaneous roles	Spinal cord injury	Circ0000381 [212]	LPS‐treated rat microglial cells	miR‐423‐3p/NLRP3	Inhibit
	Spinal cord injury/BMSCs‐derived exosome	Circ_003564 [211]	H_2_O_2_ treated rat primary neurons	NLRP3	Inhibit
	AKI/USCs‐derived exosome	Circ DENND4C [214]	Hypoxia/reoxygenation‐treated HK‐2 cells	miR‐138‐5p/FOXO3a/NLRP3	Inhibit
	Renal fibrosis	CircACTR2 [213]	PMA‐treated human monocytic leukaemia cell line THP‐1, proximal tubular epithelial cells HK‐2	miR‐561/NLRP3	Aggravate
	LUAD/Radiotherapy	CircNEIL3 [216]	Radiation‐treated A549 lung cancer cells	miR‐1184/PIF1/AIM2	Inhibit
	Silicosis	CircRNA11:120406118|12040782 [217]	Silica‐stimulated macrophages and fibroblasts	miR‐30b‐5p/NLRP3	Aggravate
	ALI	Circ‐CARD8 [215]	PMA and LPS‐treated THP‐1 cells	Hsa‐miR‐580‐3p/CARD8/caspase‐1	Inhibit
	NAFLD	CircSOD2 [218]	Palmitic acid‐treated hepatocytes	miR‐532‐3p/TXNIP/NLRP3	Aggravate

### 4.2 Noncanonical Pathway

Recent studies have begun to elucidate the regulatory roles of circRNAs in noncanonical pyroptotic pathways, particularly within arterial system pathologies [219], inflammatory disorders [220, 221]. Unlike canonical pyroptosis mediated by caspase‐1 via inflammasomes, noncanonical pathways involve alternative activators (e.g., caspase‐4/5 in humans, caspase‐11 in mice) and exhibit distinct pathological associations. These findings suggest that circRNAs may serve as modulators of noncanonical pyroptosis.

#### 4.2.1 Arterial System Pathologies

Evidence also indicates that circRNAs can modulate MI [165] pathogenesis by regulating noncanonical pyroptotic pathways. Experimental studies demonstrate that upregulated circ‐NNT in A/R‐treated cardiomyocytes and I/R‐injured murine myocardium functions as a molecular sponge for miR‐33a‐5p, thereby derepressing USP46 expression [165]. This molecular cascade culminates in the concurrent activation of caspase‐1 (canonical) and caspase‐11 (murine noncanonical) pathways, ultimately driving pyroptosis [165].

#### 4.2.2 Inflammatory Disorders

Complementing these findings in vascular disorders, recent mechanistic studies elaborate circRNAs‐mediated regulation of noncanonical pyroptotic pathways in inflammatory pathologies, with miRNA sponging mechanisms being significant in AP and periodontitis. Notably paralleling observations by Feng [177] and Wang et al. [221] revealed elevated circHIPK3 levels in serum from AP patients and caerulein‐stimulated AR42J cells. Both studies identified miR‐193a‐5p as the functional target through ceRNA function [177, 221]. However, Wang et al. further demonstrated that the circHIPK3/miR‐193a‐5p axis regulates both caspase‐1 (canonical) and caspase‐11 (noncanonical) activation, with mechanistic validation showing miR‐193a‐5p directly suppresses GSDMD expression via complementary binding to its mRNA [221]. In periodontal inflammation models, circ_0138959 was upregulated in LPS‐treated HGFs and clinical specimens from periodontitis patients [220]. Functional analysis revealed circ_0138959 promotes CASP5 expression by sponging miR‐527, thereby enhancing noncanonical pyroptosis. CircRNAs associated with noncanonical pyroptosis regulation are summarised in Table 2.

**TABLE 2:** Summary of circRNAs associated with the noncanonical pyroptosis pathway.

Classification of diseases	Diseases/Therapies	CircRNAs	Cells	Functions	Effect on pyroptosis
Arterial system pathologies	MI	Circ‐NNT [165]	A/R‐treated cardiomyocytes	miR‐33a‐5p/USP46/caspase‐1 and caspase‐11	Aggravate
Inflammatory disorders	AP	CircHIPK3 [221]	Caerulein‐treated AR42J cells	miR‐193a‐5p/caspase‐1 and caspase‐11	Aggravate
	Periodontitis	Circ_0138959 [220]	LPS‐treated HGFs	miR‐527/caspase‐5	Aggravate

### 4.3 Caspase‐3/8‐Mediated Pathway

Current reports indicate that circRNAs participate in disease pathogenesis through caspase‐3/8‐mediated pyroptosis, with most studies specifically converging on the caspase‐3/GSDME axis. Importantly, these investigations predominantly focus on tumour‐related contexts through protein interaction mechanisms. The narrow disease spectrum underscores that this research field remains at an early exploratory stage.

In LUAD, both circRPPH1 [157] and circPIBF1 demonstrated significant upregulation. These circRNAs exert their oncogenic functions through distinct protein interaction mechanisms. Mechanistically, circRPPH1 forms a complex with the transcription factor MAFK to enhance SIRT1 expression, thereby suppressing caspase‐3/GSDME‐mediated pyroptosis. The growth‐inhibitory effects of circRPPH1 knockdown (sh‐circRPPH1) on LUAD cells were significantly attenuated by SIRT1 or MAFK overexpression [157]. Additionally, circPIBF1 interacts with Nrf2, facilitating the recruitment of histone acetyltransferase E1A binding protein p300 (EP300) to the superoxide dismutase 2 (SOD2) promoter [158]. This interaction enhances H3K27 acetylation at the SOD2 promoter, resulting in upregulated SOD2 expression. Genetic silencing of circPIBF1 markedly reduced EP300 binding to the SOD2 promoter, concomitant with decreased SOD2 expression and suppressed LUAD cell proliferation. Notably, circPIBF1 knockdown also enhanced the expression of pyroptotic factors and inhibited tumour growth [158]. Moreover, emerging studies suggest that circRNA‐regulated pyroptosis contributes to osimertinib resistance in LUAD. Hsa_circ_0007312 shows significantly higher expression in tumour tissues than in normal counterparts [222]. Clinical analysis revealed that LUAD patients with low Hsa_circ_0007312 expression exhibit longer disease‐free survival and overall survival compared to those with high expression levels [222]. Importantly, hsa_circ_0007312 expression positively correlates with osimertinib half‐maximal inhibitory concentration, indicating its involvement in drug resistance. Mechanistic studies demonstrate that hsa_circ_0007312 functions through the miR‐764/MAPK1 axis. This regulatory axis suppresses caspase‐3/GSDME‐mediated pyroptosis, thereby promoting osimertinib resistance in LUAD cells [222].

Interestingly, mitochondria‐derived circRNA have been identified as protein scaffolds mediating oesophageal squamous cell carcinoma (ESCC) progression. Studies have documented that tumour cells preferentially utilise glycolysis for energy production even under normoxic conditions (Warburg effect) [223]. Consequently, rapidly proliferating malignant cells usually exhibit hypoxic conditions. Hypoxia‐inducible factor 1α (HIF‐1α) serves as the central regulator of this tumour‐associated hypoxia [159]. CircPUM1 expression showed a positive correlation with HIF‐1α accumulation in CoCl_2_‐induced hypoxic‐like conditions. Both in vitro and in vivo analyses confirmed that circPUM1 enhances ESCC tumourigenicity, with clinical data revealing an inverse correlation between circPUM1 expression levels and patient survival rates. Mechanistically, circPUM1 serves as a protein scaffold facilitating the interaction between UQCRC1 and UQCRC2—a core dimer for mitochondrial complex III assembly [159]. This scaffold function stabilises mitochondrial complex III, thereby enhancing oxidative phosphorylation and ATP production in ESCC cells. This redundancy ATP suppresses AMPK activity, ultimately inhibiting GSDME cleavage. Collectively, these findings position circPUM1 as a promising therapeutic target for ESCC through its regulatory role in mitochondrial energetics and pyroptosis pathways [159].

Beyond LUAD and ESCC, caspase‐3/GSDME‐mediated pyroptosis regulated by circRNAs has been reported to contribute to chemoresistance in colorectal cancer (CRC). Through integrated analysis of transcriptomic profiling of oxaliplatin (OXA)‐sensitive and ‐resistant tissues alongside CRC specimens and adjacent normal tissues, Lin et al. identified consistent upregulation of hsa_circ_0002891 (circPDIA3) in both OXA‐sensitive tumours and CRC tissues compared to normal counterparts [150]. Functional studies demonstrated that circPDIA3 knockdown significantly restored OXA sensitivity in chemoresistant CRC cells. Mechanistically, circPDIA3 could interact with the GSDME‐C in a direct binding manner, thereby potentiating its autoinhibitory function through blocking ZDHHC3‐ and ZDHHC17‐mediated palmitoylation. This molecular interaction effectively suppressed pyroptotic cell death. Furthermore, a novel positive feedback loop involving the circPDIA3/miR‐449a/XBP1 axis that sustains chemotherapy resistance in CRC progression was elucidated [150]. CircRNAs associated with caspase‐3/8‐mediated pyroptosis regulation are summarised in Table 3, with all of these studies reported in cancer.

**TABLE 3:** Summary of circRNAs associated with the caspase‐3/8‐mediated pyroptosis pathway.

Classification of diseases	Diseases/Therapies	CircRNAs	Cells	Functions	Effect on pyroptosis
Cancer	LUAD	CircRPPH [157]	LUAD cell	MAFK/SIRT1/caspase‐3 (circRNA–protein interactions)	Inhibit
	LUAD	CircPIBF1 [158]	LUAD cell	Nrf2/EP300/SOD2/caspase‐3 (circRNA–protein interactions)	Inhibit
	Osimertinib‐resistant lung adenocarcinoma	Hsa_circ_0007312 [222]	EGFR‐TKI‐sensitive and resistant lung adenocarcinoma	miR‐764/MAPK1/caspase‐3	Inhibit
	ESCC	CircPUM1 [159]	CoCl2‐induced intracellular hypoxic‐like condition in ESCC cell	Scaffold for the interaction between UQCRC1 and UQCRC2/caspase‐3 (circRNA–protein interactions)	Inhibit
	Chemoesistant colon cancer to OXA	CircPDIA3 [150]	CRC cell and normal colon cell	GSDME‐C (circRNA–protein interactions)	Inhibit

## 5 Conclusions and Perspectives

In this review, the regulation of canonical, noncanonical, along with caspase‐3/8‐mediated pyroptosis pathways by circRNAs is synthesised. While circRNAs are well‐established regulators of proliferation, apoptosis, autophagy and migration, their role in pyroptosis remains underexplored. Currently, the majority (approximately five‐sixths) of studies focus on the caspase‐1/GSDMD‐mediated classical pyroptosis pathway, whilst the regulation of pyroptosis mediated by caspase‐3 and caspase‐11 through circRNAs has been revealed in a limited number of articles. Furthermore, it remains unclear whether caspase‐4/8‐mediated pyroptosis can be regulated by circRNAs. Moreover, thoughtfully, while compelling evidence establishes that circRNAs regulate apoptotic pathways through caspase‐3/8 modulation, a critical question arises regarding studies investigating circRNA‐mediated caspase‐3/8 control in GSDME/C‐highly expressed cellular models: Could pyroptosis activation confound observed apoptotic outcomes? Additionally, the discovery that granzymes cleave gasdermin family members challenges the conventional understanding that pyroptosis initiation depends on caspases [14, 15]. While emerging evidence indicates that circRNAs can regulate granzyme secretion [224, 225, 226, 227, 228], current studies have not yet explored the consequential effects on pyroptosis. Elucidating the mechanistic link between circRNA‐mediated granzyme modulation and pyroptotic cell death represents a critical knowledge gap that warrants further investigation. Moreover, reports indicate that chronic pyroptosis in the hypoxic tumour core, mediated by a small subset of tumour cells, suppresses antitumour immunity and accelerates tumour progression [5]. In contrast, acute pyroptotic responses demonstrate immunostimulatory effects that inhibit tumour growth. Current studies on circRNA‐regulated pyroptosis predominantly focus on determining the presence or absence of this cellular process, while insufficient attention has been given to how its induction or suppression modulates antitumour immunity. The biological consequences of pyroptosis induction or suppression in tumour cells require comprehensive investigation regarding their immunomodulatory and tumour microenvironmental remodelling potential. It is noteworthy that although circRNAs function through diverse mechanisms, current research on their regulation of pyroptosis predominantly focuses on the miRNA sponge mechanism. This preference is largely attributed to the straightforward logic of sequence‐based function, well‐established experimental methodologies and databases. However, several limitations exist in miRNA sponge‐dependent circRNA‐mediated pyroptosis regulation. One major concern is the dose dependency between circRNAs and miRNAs. Under physiological conditions, most circRNAs are expressed at low levels, and even several‐fold changes under pathological conditions may not result in biologically meaningful differences in their expression levels. Therefore, many studies that rely on circRNAs overexpression to induce marked downstream effects warrant more cautious interpretation. In addition, the dual‐luciferase reporter assay, which accounts only for the primary RNA sequence while neglecting higher‐order structures, substantially reduces the accuracy of ceRNA functional validation. Due to these methodological limitations, further verification is needed for studies investigating circRNA‐mediated regulation of pyroptosis through the ceRNA mechanism. Fortunately, accumulating evidence has demonstrated that circRNAs can regulate pyroptosis by directly interacting with proteins [150, 151, 152, 153, 154, 155, 156, 157, 158, 159], underscoring their critical role in post‐transcriptional regulation. Notably, circPDIA3 binds directly to GSDME‐C, enhancing the autoinhibitory function of its C‐terminal domain. Such direct circRNA–protein interactions may help minimise off‐target effects [150]. Emerging evidence further reveals the coding potential of circRNAs, with the cryptically translated peptide CDC42‐165aa demonstrating functional significance in pyroptosis regulation [160]. The potential involvement of uncharacterised circRNA‐encoded proteins in pyroptosis warrants in‐depth investigation to advance our understanding of this critical biological process. Furthermore, the remarkable success of mRNA vaccines in combating the COVID‐19 pandemic [229, 230, 231, 232] has accelerated the development of circRNA‐based vaccine platforms. Compared with linear mRNA vaccines, circRNA vaccines demonstrate superior molecular stability, prolonged protein expression and enhanced immunogenicity, making them particularly promising candidates [233, 234]. Preclinical successes have been achieved with circRNA vaccines targeting COVID‐19 [235, 236], monkeypox virus [237] and malignancies [238]. Meanwhile, pharmacological targeting of GSDMD has shown therapeutic potential in sepsis [207, 208], MI [208, 239] and tumour [240] based on robust experimental evidence. This raises a critical research direction: Can circRNA‐based therapeutic platforms be engineered to selectively induce tumour cell pyroptosis while suppressing pathological pyroptosis in normal cells, thereby developing novel treatment strategies for cancer and other diseases?

With expanding research on pyroptosis, this cell death mechanism has been defined as a lytic, proinflammatory, and programmed form of cell death. The breakthrough discovery of gasdermins as pyroptosis executors has transformed our understanding in this field. However, significant gaps remain in our understanding of the biological functions of other gasdermins family members, particularly their potential roles in pyroptosis regulation and associated phenotypic manifestations. Historically, PMR following cellular swelling was considered a passive process. However, recent studies demonstrate that GSDMD pore formation alone is insufficient to induce PMR, which is essential for pyroptosis execution. Notably, PMR is actively mediated by NINJ1, a 16 kDa membrane protein. NINJ1 is required for the release of DAMPs and lactate dehydrogenase following GSDMD activation. Pyroptotic cells lacking NINJ1 may fail to amplify inflammatory responses [17, 241, 242]. While emerging as a pivotal player in regulated cell death, critical gaps remain regarding NINJ1 biology, particularly the triggers for its oligomerisation and the pathophysiological consequences of NINJ1‐mediated membrane disruption in inflammatory progression and disease pathogenesis. Additionally, gasdermin proteins amplify tumour immunity by inducing pyroptosis in a small number of cancer cells. Immune cells demonstrate the capacity to discriminate between normal and malignant cells, thereby selectively triggering enhanced tumour cell elimination while minimising drug‐related toxic effects on healthy tissues. This highlights the therapeutic significance of identifying small molecules that alleviate gasdermin's autoinhibition. CircPDIA3 has been reported to enhance GSDME‐C autoinhibition [150], suggesting that identifying circRNAs capable of disrupting gasdermin autoinhibition holds significant promise. Furthermore, whether tumour‐specific pyroptotic pathways exist and whether the cancer‐specific expression patterns of circRNAs [243] enable them to serve as tumour‐selective triggers for pyroptosis warrant further exploration.

## Author Contributions

Tengyu Jin: conceptualisation, writing – original draft. Guodong Xu: writing – original draft. Wanru Zhou: writing – original draft. Yige Shi: data curation, visualisation. Hebo Wang: conceptualisation, funding acquisition, writing – review and editing.

## Funding

This work was supported by the Major Science and Technology Support Plan of Hebei Province (Project no. 242W7703Z).

## Ethics Statement

The authors have nothing to report.

## Consent

The authors have nothing to report.

## Conflicts of Interest

The authors declare no conflicts of interest.

## Data Availability

Data sharing not applicable to this article as no data sets were generated or analysed during the current study.
